# Seclidemstat (SP-2577) Induces Transcriptomic Reprogramming and Cytotoxicity in Multiple Fusion–Positive Sarcomas

**DOI:** 10.1158/2767-9764.CRC-24-0296

**Published:** 2025-09-10

**Authors:** Galen C. Rask, Cenny Taslim, Ariunaa Bayanjargal, Rachel D. Dreher, Matthew V. Cannon, Julia Selich-Anderson, Jesse C. Crow, Aundrietta Duncan, Emily R. Theisen

**Affiliations:** 1 Center for Childhood Cancer Research, Abigail Wexner Research Institute at Nationwide Children’s Hospital, Columbus, Ohio.; 2 Medical Scientist Training Program, College of Medicine, The Ohio State University, Columbus, Ohio.; 3 Salarius Pharmaceuticals, Houston, Texas.; 4 Department of Pediatrics, College of Medicine, The Ohio State University, Columbus, Ohio.

## Abstract

Genes encoding the RNA-binding proteins FUS, EWSR1, and TAF15 (FET proteins) are involved in chromosomal translocations in rare sarcomas. The encoded fusion oncoproteins typically include a DNA-binding domain and function as oncogenic transcription factors. FET-rearranged sarcomas are often aggressive malignancies affecting patients of all ages. Oncogenic fusion transcription factors are challenging to target directly, and new therapies are needed. Treatment with the small molecule SP-2509 results in reversal of the transcriptional activity of the FET fusion that causes Ewing sarcoma, EWSR1::FLI1. A similar molecule, seclidemstat (SP-2577), is currently in clinical trials for FET-rearranged sarcomas (NCT03600649), but its pharmacologic activity in FET fusion–positive sarcomas has not been demonstrated. We found potent seclidemstat cytotoxicity against both FET-rearranged and other fusion-positive sarcoma cell lines *in vitro*, including Ewing sarcoma, desmoplastic small round cell tumor, clear cell sarcoma, myxoid liposarcoma, and fusion-positive rhabdomyosarcoma. We also define transcriptomic effects of seclidemstat with bulk RNA sequencing. Seclidemstat recapitulated much of SP-2509 transcriptional activity in Ewing sarcoma. Widespread transcriptional changes were seen in all tested cell lines after seclidemstat treatment, regardless of the fusion protein expressed. This included reversal of FET fusion transcriptional signatures for EWSR1::WT1, EWSR1::ATF1, and EWSR1::ERG. Though novel inhibitors are unlikely to display single-agent efficacy in the clinic, these data suggest that seclidemstat remains a promising new treatment strategy for patients with FET-rearranged and other fusion-positive sarcomas.

**Significance:**

In this study, we show seclidemstat has *in vitro* activity in multiple rare and aggressive sarcomas caused by FET fusion proteins. With 13 RNA sequencing experiments, including multiple FET-rearranged sarcoma cell lines, this dataset is a rich resource for those studying FET-rearranged sarcomas.

## Introduction

Genes encoding the RNA-binding proteins FUS, EWSR1, and TAF15 (FET family proteins) are frequently involved in chromosomal translocations in sarcomas (1). The resulting fusion genes typically involve a FET family gene in the 5′-portion of the fusion and a gene encoding a transcription factor (TF) in the 3′-portion of the fusion (1, 2). The proteins expressed from these fusions include the amino-terminal intrinsically disordered and low-complexity domain (LCD) of the FET protein and a carboxyterminal DNA-binding domain (DBD; refs. 1, 2). The LCD functions as a strong transcriptional activator, and these chimeras function as oncogenic TFs. Sarcomas with FET rearrangements are thus often characterized by a relatively low mutational burden and widespread deregulation of chromatin and transcription (2–11).

The epidemiology of tumors involving FET rearrangements varies. For example Ewing sarcoma, involving FET::ETS fusions (primarily EWSR1::FLI1), is predominantly a tumor of adolescents and young adults (12). This is an aggressive malignancy, and though patients with local disease have ∼70% 5-year survival, patients with metastatic, relapsed, and refractory disease have dismal outcomes with 10% to 30% survival (13, 14). Desmoplastic small round cell tumor (DSRCT) is a rare and aggressive mesenchymal tumor characterized by the expression of the EWSR1::WT1 fusion (15). DSRCT primarily affects adolescents and young adults and has extremely poor outcomes, with ∼60 to 70% of patients succumbing to disease within 3 years of diagnosis (15). Clear cell sarcoma is characterized by expression of EWSR1::ATF1 fusion protein and has a peak incidence in middle age (16). Despite multimodal therapy, the 5-year survival is ∼50% and 10-year survival is ∼38% (16). Myxoid liposarcomas represent ∼30% of all liposarcomas and are characterized by the expression of the FUS::DDIT3 fusion (17). Peak incidence for this disease is in adults near 50 years old, and its 5-year survival is ∼75 to 85% (17). Outcomes are worse for those with metastatic or high-grade disease (17). Outcomes for patients with metastatic and relapsed FET-rearranged sarcomas have remained unchanged for decades, and new therapeutics options are urgently needed.

As the causative oncogenes, these fusion proteins make attractive therapeutic targets. Unfortunately, the intrinsically disordered nature of the LCD and the convex binding surface of TF DBDs make pharmacologically targeting these proteins difficult (12). Instead, inhibiting the critical regulators and coregulators of the fusion protein has emerged as an attractive therapeutic strategy (18–23). Toward this end, many chromatin coregulators have been identified that interact with the N-terminal FET domain, including the ATP-dependent chromatin remodeling BAF complex (2, 7, 10, 24), the histone acetyltransferase p300 (9), histone deacetylases (HDAC; ref. 25), and the histone lysine demethylase LSD1 (18, 24, 26). Our prior results showed that treatment with the noncompetitive LSD1 inhibitor, SP-2509, reverses the transcriptional activity of both EWSR1::FLI1 and EWSR1::ERG in Ewing sarcoma (18). Notably, whereas treatment with SP-2509 interfered with both fusion-dependent gene activation and repression, HDAC inhibition only disrupted EWSR1::FLI1–mediated repression (18).

Combined with results showing single-agent activity in xenograft models (18), these data spurred the development of a clinical lead compound with improved physicochemical and pharmacokinetic properties, seclidemstat (SP-2577; refs. 27–29). Seclidemstat is currently in clinical trials for Ewing sarcoma and related FET-rearranged tumors (NCT03600649 and NCT05266196), as well as myelodysplastic syndrome and chronic myelomonocytic leukemia (NCT04734990). Though seclidemstat is structurally similar to SP-2509, its effects on the transcriptional activity of EWSR1::FLI1 have not been directly assessed, and it is unknown whether its pharmacodynamic effects are comparable with SP-2509. Additionally, given reported similarities in the fusion-driven transcriptional changes in different FET-rearranged tumors (11) and the demonstrated recruitment of LSD1 by multiple FET fusion proteins (24, 26) treatment with seclidemstat may also alter the transcriptional activity of other fusion proteins. We therefore tested the pharmacodynamic effects of seclidemstat treatment on the transcriptome of varied FET and non-FET fusion oncoproteins in *in vitro* models of multiple malignancies: Ewing sarcoma, DSRCT, clear cell sarcoma, myxoid liposarcoma, and fusion-positive rhabdomyosarcoma (FP-RMS). These studies revealed that all disease models were sensitive to treatment with noncompetitive LSD1 inhibitors and that seclidemstat treatment disrupted FET fusion activity in all the cell lines tested, as seen for SP-2509 in Ewing sarcoma.

## Materials and Methods

### Reagents and constructs

SP-2509 and OG-L002 were purchased from Selleck Chemicals (cat. #S7680 and S7237, respectively). Seclidemstat was provided by Salarius Pharmaceuticals. SP-2513 was synthesized by the Medicinal Chemistry Shared Resource at The Ohio State University James Comprehensive Cancer Center. Identity and purity of SP-2513 were verified by high-performance liquid chromatography–mass spectrometry and nuclear magnetic resonance. The luciferase RNAi (iLuc), pMSCV-empty vector (hygro), and pMSCV-3X-FLAG EWSR1::FLI1 (hygro) are previously described (30, 31). The EWSR1::ERG RNAi construct is previously described (30), as is the pMSCV 3X-FLAG EWSR1::ERG construct (30).

### Cell lines and tissue culture

Ewing sarcoma cell lines A673 (RRID: CVCL_0080), TTC-466 (RRID: CVCL_A444), SK-N-MC (RRID: CVCL_0530), and TC32 (RRID: CVCL_7151) were provided by Dr. Stephen Lessnick. The DSRCT cell lines JN-DSRCT-1 (RRID: CVCL_9W68) and BER (RRID: CVCL_E479) were kindly provided by Dr. Sean B. Lee (Tulane University). The myxoid liposarcoma cell lines 1765-92 (RRID: CVCL_S817) and 402-91 (RRID: CVCL_S813), as well as the clear cell sarcoma cell line DTC1 (RRID: CVCL_0J31), were kindly provided by Dr. Torsten Nielsen (University of British Columbia). The clear cell sarcoma cell line SU-CCS-1 (RRID: CVCL_B470) was purchased from the ATCC (#CRL-2971). DL221 was provided by Salarius Pharmaceuticals. The FP-RMS cell lines RH4 (RRID: CVCL_5916), RH30 (RRID: CVCL_0041), and CW9019 (RRID: CVCL_N820) were provided by Dr. Benjamin Stanton (Nationwide Children’s Hospital). All cells were maintained at 37°C, 5% CO_2_. A673 cells were cultured in DMEM (Corning Cellgro, 10-013-CV) supplemented with 10% FBS (Gibco, 16000-044), penicillin/streptomycin/glutamine (P/S/Q; Gibco, 10378-016), and sodium pyruvate (Gibco, 11360-070). DL221 and the FP-RMS cells were cultured in DMEM supplemented with 10% FBS and P/S/Q. JN-DSRCT-1 and BER cells were grown in DMEM/F12 media with 10% FBS and P/S/Q. All of the remaining cell lines (TTC-466, 1765-92, 402-91, SU-CCS-1, and DTC1) were cultured in RPMI with 10% FBS and P/S/Q. All experiments were performed within 2 months of thawing a cell line. All cell lines identities were verified using short tandem repeat profiling (LabCorp) upon thawing and are checked annually. Additional confirmation of appropriate fusion gene expression was confirmed using EnFusion as described below. Cells were tested for *Mycoplasma* using the Universal Mycoplasma Detection Kit (ATCC, 30-1012K) and are checked annually.

### Retrovirus production and transduction of TTC-466 cells

Viral production using HEK-293 (RRID: CVCL_6974) cells was performed as previously described (18). short hairpin RNAs (shRNA) and cDNAs were transduced on day 1, with cDNA transduction beginning 4 hours following shRNA transduction. Transduction was performed with viral supernatants containing 2.5 μL of 8 μg/mL polybrene. Plain media were added overnight, and on day 3, cells were passaged into selection media containing 2 μg/uL puromycin (Sigma-Aldrich, P8833) and 150 μg/mL hygromycin B (Thermo Fisher Scientific, 10687010) for 10 days. Following 10 days, cells were seeded into soft agar assays and collected for RNA and protein isolation.

### Antibodies

The following antibodies were used for immunodetection: M2-anti-FLAG (Sigma-Aldrich, F3165, RRID: AB_259529), anti-ERG (Abcam ab92513, RRID: AB_2630401), anti–α-tubulin (DM1A; Abcam ab7291, RRID: AB_2241126), IRDye 800CW goat anti-mouse IgG (LI-COR Biosciences, 926-32210, RRID: AB_621842), and IRDye 800CW goat anti-rabbit IgG (LI-COR Biosciences, 926-32211, RRID: AB_621843).

### Cell viability assays

Cell viability was assessed by seeding cells in 96-well white, opaque, tissue culture–treated plates (Corning) at a density of 10,000 cells per well in 200 μL of corresponding media. After 24 hours on the plates, cells were treated independently with a 9-point 3.3× increasing dosing scheme of SP-2509, seclidemstat, SP-2513, and OG-L002 (2 nmol/L–30 μmol/L). After 96 hours, 122 μL of media were removed from each well and 80 μL of CellTiter-Glo (Promega) was added to each well. The plates were agitated while covered at 250 rpm for 10 minutes at room temperature. Luminescence was measured on a Synergy H1 plate reader (Agilent). Cell viability was calculated relative to vehicle-treated wells.

### Protein isolation and Western blot

Total cellular protein was extracted from frozen cell pellets using the Pierce RIPA buffer (Thermo Fisher Scientific, 88901) with protease inhibitor cocktail added (Sigma-Aldrich, P8340). Pellets were resuspended in RIPA for 30 minutes, vortexed vigorously every 10 minutes, and centrifuged at maximum speed for 30 minutes at 4°C. Protein concentration was determined using the Pierce BCA Protein Assay Kit (Thermo Fisher Scientific, 23225). Fifteen μg of protein sample was run on precast 4% to 15% gradient Tris-glycine gels (Bio-Rad, 4561084) at 90 V for 15 minutes and 120 V for 60 minutes. Proteins were transferred to nitrocellulose membranes (Thermo Fisher Scientific, IB23002) using the iBlot 2 (Thermo Fisher Scientific, IB21001). Membranes were blocked with Odyssey PBS Blocking Buffer (LI-COR Biosciences, 927-40003) for 1 hour at room temperature. Immunoblotting was performed with primary antibody overnight at 4°C.

### Soft agar assays

Soft agar assays were performed as previously described (25, 32, 33), with 18,000 cells/plate seeded for TTC-466 cells.

### RNA isolation and RNA sequencing submission

Cells were seeded with 1 × 10^6^ cells in 10 mL media per 10-cm dish for RNA sequencing (RNA-seq) experiments. After 24 hours, media were replaced with fresh media containing either vehicle (0.3% DMSO), the IC_90_ of seclidemstat as outlined in Table 1, or 2 μmol/L SP-2509. Cells were treated for 48 hours, trypsinized, washed in 1X Hank’s Balanced Salt Solution, pelleted, and flash-frozen for RNA extraction.

**Table 1:** FET-rearranged cell line models and RNA-seq dosing used for this study

Tumor	Cell line	Fusion	Seclidemstat dose for RNA-seq (nmol/L)
Ewing sarcoma	A673	EWSR1::FLI1	1,076
	TC32	EWSR1::FLI1	N/A
	SK-N-MC	EWSR1::FLI1	N/A
	TTC-466	EWSR1::ERG	1,069
DSRCT	JN-DSRCT-1	EWSR1::WT1	1,265
	BER	EWSR1::WT1	2,546
Clear cell sarcoma	SU-CCS-1	EWSR1::ATF1	802.2
	DTC1	EWSR1::ATF1	2,758
Myxoid liposarcoma	1765-92	FUS::DDIT3	2,766
	402-91	FUS::DDIT3	1,452
	DL221	FUS::DDIT3	14,091

Total mRNA was extracted from cell pellets following the manufacturer’s instructions of the RNeasy kit (Qiagen), including the use of a genomic DNA removal column. RNA concentration was quantified using a NanoDrop device, and 1 μg of RNA per sample was submitted to the Abigail Wexner Research Institute Genomic Services Laboratory (RRID: SCR_017840) for library preparation using the TruSeq Stranded mRNA Kit (Illumina 20020594), library quality control (QC), and next-generation sequencing using an Illumina NovaSeq 6000 (RRID: SCR_016387). Samples were sequenced with a targeted sequencing depth of 50 million 150-bp paired end reads per sample. Raw BCL files were converted to FASTQ files and returned following the sequencing runs.

### RNA-seq data analysis

Publicly available data from FASTQ files were analyzed using an in-house containerized pipeline that performs QC [FastQC (34) and MultiQC (35)], adapter and low-quality read trimming [TrimGalore! (36), RRID: SCR_011847], alignment to hg38 reference genome [STAR 2.6 (37)], and assignment to genomic features [featureCounts (38), RRID: SCR_012919] and analyzed for differential expression [DESeq2 (39), RRID: SCR_015687; SARTools (40), RRID: SCR_016533]. Fusion detection was run using EnFusion as described (41). Overlap analysis was done using GeneOverlap (RRID: SCR_018419; ref. 42) and visualized using eulerr (RRID: SCR_022753; ref. 43), venn (44), and UpSetR (RRID: SCR_026112; ref. 45). Uniform Manifold Approximation and Projection (UMAP) was plotted using the R umap package (RRID: SCR_018217; ref. 46). Other visualization was done with ggplot2 (RRID: SCR_014601; ref. 47) and base R packages. Pathway analysis was performed using clusterProfiler (RRID: SCR_016884; ref. 48) and MSigDB database (RRID: SCR_016863; ref. 49). Gene set enrichment analysis (GSEA; RRID: SCR_003199) was performed as described with direct target gene sets used in GSEA for EWSR1::FLI1 (50), EWSR1::WT1 (51), and EWSR1::ATF1 (6) were described previously. Briefly, for all three fusions direct target genes were defined as those genes (i) near a fusion protein–bound locus with characteristics of active chromatin or fusion-mediated chromatin loop and (ii) with differential expression upon genetic depletion of the fusion.

### Statistical analysis

Dose–response curves for cell viability data were determined in GraphPad Prism 9 (RRID: SCR_002798) using the 4-parameter, variable slope log(inhibitor) vs. response equation. Three technical replicates were included in each of two biological replicates. Differentially expressed genes (DEG) were defined as genes with a multiple hypothesis testing adjusted [FDR/Benjamini–Hochberg (52)] *P* value of < 0.05. Statistical significance of Venn overlap analysis was evaluated using the Jaccard index (53, 54), which measures the similarity and Fisher exact test for statistical significance (55). GSEA was performed as described (56), with normalized enrichment scores (NES) and *P* values reported.

### Data availability

The data sets analyzed in this work are deposited in the NCBI’s Gene Expression Omnibus and Sequencing Read Archive and are accessible through Gene Expression Omnibus Series Accession GSE267611. All other data are available within the main manuscript, supplemental files, or upon request from the corresponding author.

## Results

### Noncompetitive LSD1 inhibitors show potent activity against cell lines derived from FET-rearranged tumors

In order to determine the transcriptional effects of seclidemstat treatment in different FET-rearranged cell lines, we first sought to define the potency of seclidemstat against cell lines expressing these fusions in cell viability assays. We used the following cell lines: Ewing sarcoma—A673, TC32, SK-N-MC, and TTC-466; DSRCT—JN-DSRCT-1, and BER; clear cell sarcoma—SU-CCS-1 and DTC1; and myxoid liposarcoma—1765-92, 402-91, DL221. The respective fusions expressed in each cell line are shown in Table 1. In addition to seclidemstat, we included SP-2509 for comparison with another noncompetitive LSD1 inhibitor as well as OG-L002 for comparison with an irreversible LSD1 inhibitor with similar potency and specificity (57). Lastly, we included a compound structurally similar to SP-2509 with no activity against LSD1, SP-2513 [cpd 13 (58)], to exclude off-target cytotoxic effects caused by the hydrazide moiety in seclidemstat and SP-2509.

**Figure 1:**
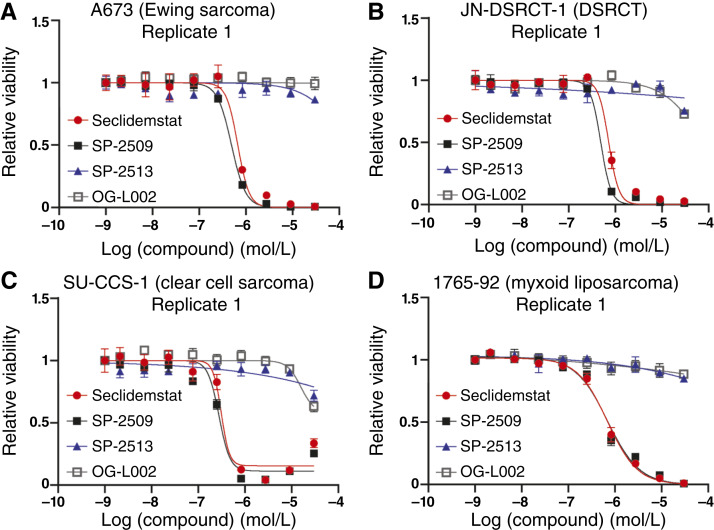
Noncompetitive LSD1 inhibitors show potent activity in cell viability assays using FET-rearranged sarcoma cell lines. **A–D,** Dose–response curves for seclidemstat (red/circle), SP-2509 (black/closed square), SP-2513 (blue/triangle), and OG-L002 (gray/open square) in (**A**) A673, (**B**) JN-DSRCT-1, (**C**) SU-CCS-1, and (**D**) 1765-92 cells. Data are displayed for a single biological replicate. Mean values of three technical replicates are shown with SD. Calculated curves of best fit are also shown.

As previously reported (32), all Ewing sarcoma cell lines were sensitive to treatment with SP-2509, with IC_50_ values ranging from 30 to 500 nmol/L, and resistant to treatment with OG-L002, with no determinable IC_50_ value up to 30 μmol/L (Fig. 1A; Table 2; Supplementary Fig. S1). Ewing sarcoma cells were also sensitive to seclidemstat with IC_50_ values ranging from 290 to 700 nmol/L (Fig. 1A; Table 2; Supplementary Fig. S1). This slight decrease in *in vitro* potency is expected. The structure of SP-2509 contains a morpholine ring, which promotes cell permeability, whereas seclidemstat instead possesses an N-methylpiperazine (27, 58). This moiety improves formulation, solubility, oral bioavailability, and pharmacokinetics but can reduce permeability in tissue culture assays. Notably, SP-2513 showed no activity in cell viability assays, with IC_50_ values not determinable below 30 μmol/L (Fig. 1A; Table 2; Supplementary Fig. S1).

**Table 2:** IC_50_ values for dose–response curves in FET-rearranged tumor cell lines

Disease	Fusion	Cell line	SP-2509				SP-2577			
			Replicate 1		Replicate 2		Replicate 1		Replicate 2	
			IC_50_ (nmol/L)	95% CI (nmol/L)	IC_50_ (nmol/L)	95% CI (nmol/L)	IC_50_ (nmol/L)	95% CI (nmol/L)	IC_50_ (nmol/L)	95% CI (nmol/L)
EWS	EWSR1::FLI1	A673	493	468–520	277	248–312	671	603–748	571	449–ND
		TC32	51.7	46.3–57.5	172	154–192	292	261–333	369	341–394
		SK-N-MC	483	409–574	80.1	65.7–95.2	511	446–591	376	322–427
	EWSR1::ERG	TTC-466	69.7	49.3–95.6	31.6	22.7–42.6	325	289–366	322	283–369
DSRCT	EWSR1::WT1	JN-DSRCT-1	487	447–527	420	371–476	722	649–775	504	322–712
		BER	1,040	953–1,180	363	284–461	1,430	1,320–1,560	1,110	ND–1,370
CCS	EWSR1::ATF1	SU-CCS-1	279	249–330	82.5	76.9–94.6	313	286–443	265	233–309
		DTC1	1,120	ND-1,340	1,380	1,150–1,700	1,390	1,230–1,570	1,580	1,410–1,770
MLS	FUS::DDIT3	1765-92	692	585–821	511	438–595	690	616–774	480	301–781
		402-91	821	653–1,010	620	533–ND	1,080	ND–1,170	630	550–ND
		DL221	7,450	4,850–12,300	4,390	3,130–6,330	4,650	3,650–6,190	3,890	3,040–5,050

​	​	Cell line	SP-2513				OG-L002			
			Replicate 1		Replicate 2		Replicate 1		Replicate 2	
			IC_50_ (nmol/L)	95% CI (nmol/L)	IC_50_ (nmol/L)	95% CI (nmol/L)	IC_50_ (nmol/L)	95% CI (nmol/L)	IC_50_ (nmol/L)	95% CI (nmol/L)
All	All	All tested	ND	N/A	ND	N/A	ND	N/A	ND	N/A

Compiled IC_50_ values for SP-2509, seclidemstat, SP-2513, and OG-L002 for all tested cell lines and replicates. IC_50_ values were determined using GraphPad Prism 9 and are reported with the 95% confidence interval.

Abbreviations: CCS, clear cell sarcoma; CI, confidence interval; EWS, Ewing sarcoma; MLS, myxoid liposarcoma; ND, not determined.

A similar pattern of activity emerged in DSRCT and clear cell sarcoma. IC_50_ values for SP-2509 and seclidemstat were 300 nmol/L to 1.1 μmol/L and 500 nmol/L to 1.5 μmol/L, respectively, in both DSRCT cell lines tested (Fig. 1C; Table 2; Supplementary Fig. S2A–S2C). Clear cell sarcoma cell lines had IC_50_ values ranging from 80 nmol/L to 1.4 μmol/L and 250 nmol/L to 1.6 μmol/L for SP-2509 and seclidemstat, respectively (Fig. 1D; Table 2; Supplementary Fig. S2D–S2F). IC_50_ values were undeterminable for OG-L002 and SP-2513 in these cell lines below 30 μmol/L (Fig. 1B and C; Table 2; Supplementary Fig. S2A–S2F). Similarly, neither OG-L002 nor SP-2513 showed activity in any of the myxoid liposarcoma cell lines (Fig. 1E; Table 2; Supplementary Fig. S2G–S2K). There was more variable potency observed for SP-2509 and seclidemstat in the myxoid sarcoma cell lines. 1765-92 and 402-91 had IC_50_ values ranging from 500 to 850 nmol/L for SP-2509 and 450 nmol/L to 1.1 μmol/L for seclidemstat. In contrast, the DL221 cell line was more resistant. We were unable to fit a 4-parameter dose–response curve, because there were not enough data to establish the bottom of the curve below 30 μmol/L, above which we encounter challenges related to solubility (Supplementary Fig. S2J and S2K). Instead, we fixed the bottom of the curve to 0 and used the estimated IC_50_ value. The IC_50_ values across replicates ranged from 4.2 to 7.5 μmol/L for SP-2509 and 3.8 to 4.7 μmol/L for seclidemstat.

Taken together, these data show that seclidemstat has comparable potency to SP-2509 in all the cell lines tested. Moreover, other FET-rearranged tumors showed a similar pattern of activity, with sensitivity to the noncompetitive LSD1 inhibitors but no sensitivity to the irreversible or catalytic inhibitor OG-L002. This suggests that cytotoxic activity does not require inhibition of LSD1 enzymatic activity. The exception was the DL221 myxoid liposarcoma cell line, which was somewhat resistant to noncompetitive inhibitors. Nonetheless, SP-2509 and seclidemstat showed more activity than OG-L002 and SP-2513 in this cell line. We also found that this pattern of compound activity was not specific to FET-rearranged sarcomas. We analyzed additional data for SP-2509, SP-2513, and OG-L002 in FP-RMS cell lines RH4 (PAX3::FOXO1), RH30 (PAX3::FOXO1), and CW9019 (PAX7:FOXO1). Some activity had already been reported for seclidemstat in *in vivo* models of RMS (59), and we found that FP-RMS cell lines were also sensitive to SP-2509 treatment with IC_50_ values below 1 μmol/L for all tested lines (Supplementary Fig. S3; Supplementary Table S1). Like FET-rearranged cell lines, SP-2513 and OG-L002 showed little cytotoxic activity. (Supplementary Fig. S3; Supplementary Table S1).

### Additional bioinformatic tools confirm cell line and data integrity in transcriptomic analyses

We used our calculated dose response curves to estimate the dose at which 90% of cells are killed (IC_90_) for each cell line and used this dose for our transcriptomic analyses (Table 1) to match what had been done previously with SP-2509 in Ewing sarcoma cells (18). Cells were treated for 48 hours with either the DMSO vehicle or seclidemstat prior to harvest. Cells were collected, and RNA was submitted for RNA-seq analysis. Though we regularly perform short tandem repeat profiling on all the cell lines used in the laboratory, some cell lines in this study (DTC1 and DL221) did not have reference profile publicly available. Therefore, as another layer of QC, we used the EnFusion pipeline to detect fusion transcripts expressed in each cell line from our RNA-seq data (GSE267611; ref. 41). This pipeline confirmed expression of *EWSR1::FLI1* and *EWSR1::ERG* in A673 and TTC-466 cell lines, respectively (Supplementary Fig. S4A and S4B). *EWSR1::WT1* was detected in both JN-DSRCT-1 and BER, with different breakpoints predicted for each cell line (Supplementary Fig. S4C and S4D). Both SU-CCS-1 and DTC1 were confirmed to express *EWSR1::AFT1* with the same predicted breakpoint (Supplementary Fig. S4E and S4F). Notably, despite the large differences seen between DL221 and the other two mxyoid liposarcoma cell lines, all three had detectable expression of *FUS::DDIT3* (Supplementary Fig. S4G–S4I). Because both SP-2509 and seclidemstat inhibit LSD1—and because LSD1 can be incorporated into distinct complexes with diverse functions depending on the cell type— we next examined whether any known LSD1 interactors showed significantly altered expression in DL221 cells compared with the other cell lines. Though DL221 clustered apart from other myxoid liposarcoma cell lines, it did cluster with other sensitive cell lines, and we did not find expression levels of LSD1 interactors that were associated with sensitivity (Supplementary Fig. S5). As a final step of QC and to ensure proper handling of samples and data, we used UMAP to assess all our RNA-seq samples used for analysis. We found that samples, either treated or untreated, clustered together first by disease and second by cell line (Supplementary Fig. S6). These data indicate that interdisease variability drove the largest differences in transcriptomes across samples, as expected.

### Seclidemstat treatment alters transcription in a similar manner as SP-2509 in Ewing sarcoma

**Figure 2:**
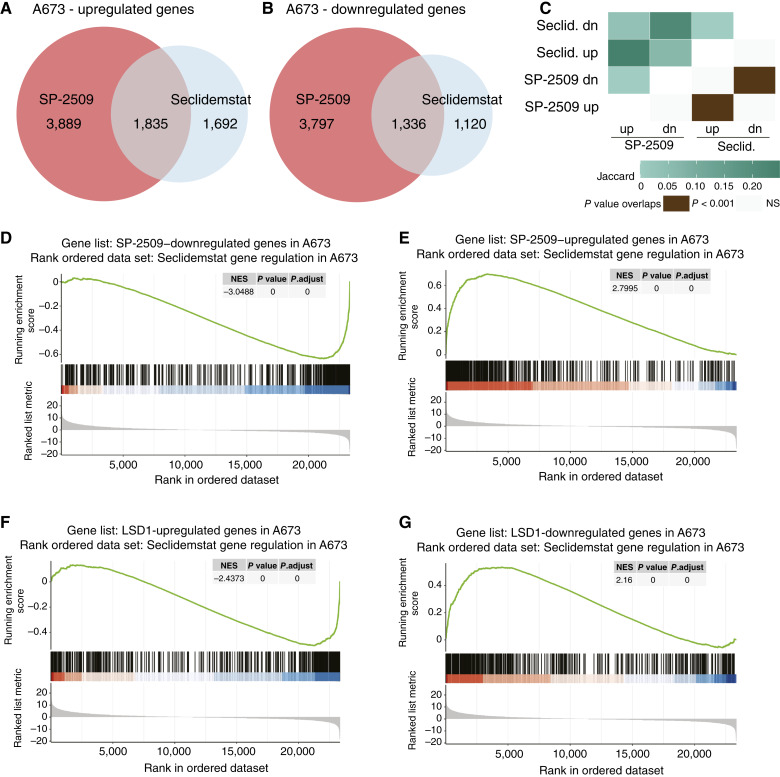
Seclidemstat transcriptional activity is similar to SP-2509 in Ewing sarcoma. **A–C,** Venn overlap analysis of SP-2509 and seclidemstat (**A**) upregulated and (**B**) downregulated genes in A673 cells with the Jaccard index and *P* values of overlap shown in **C**. dn, downregulated; Seclid., seclidemstat; NS, not significant; up, upregulated. **D–G,** GSEA of (**D**) SP-2509–downregulated, (**E**) SP-2509–upregulated, (**F**) LSD1-activated, and (**G**) LSD1-repressed genes with seclidemstat gene regulation as the rank-ordered list. NES, *P* value, and multiple hypothesis adjusted *P* values are shown in inset tables.

Having confirmed appropriate fusion expression and sample clustering, we analyzed our differential expression data for cells treated with seclidemstat. We first asked whether treatment with seclidemstat was similar to treatment with SP-2509 and how seclidemstat treatment affected LSD1-regulated and EWSR1::FLI1–regulated genes. To do this, we reanalyzed our most recent SP-2509 data from Pishas and colleagues (32) with our current RNA-seq pipeline for comparison to seclidemstat. We found that both SP-2509 and seclidemstat upregulate and downregulate the expression of thousands of genes with significant overlap in the DEGs in both treatment conditions (Fig. 2A–C). Likewise, using GSEA (56), we found significant functional similarity in seclidemstat and SP-2509 downregulated genes (NES, −3.0488; Fig. 2D) and upregulated genes (NES, 2.7995, Fig. 2E). These data demonstrate significant similarity in the transcriptional changes caused by treatment with both seclidemstat and SP-2509 in A673 Ewing sarcoma cells.

Having shown that seclidemstat largely recapitulates the transcriptional effects of SP-2509, we also wanted to determine the degree to which seclidemstat disrupts EWSR1::FLI1 transcriptional activity and LSD1 function in Ewing sarcoma cells. As for SP-2509 above, we reprocessed previously published data for EWSR1::FLI1–regulated and LSD1-regulated genes (bioRxiv 2024.01.31.578127; ref. 32) with our current pipeline. Importantly, this analysis of EWSR1::FLI1 gene regulation uses only DEGs detected upon rescue with EWSR1::FLI1 in a knockdown (KD)–rescue experiment. This restricts analysis to genes regulated by EWSR1::FLI1 expression and excludes genes whose expression changes as an off target of RNAi-mediated KD. Four-way Venn overlaps for SP-2509, seclidemstat, EWSR1::FLI1, and LSD1 DEGs show significant overlaps in genes downregulated by seclidemstat and upregulated by both LSD1 and EWSR1::FLI1 and *vice versa* (Supplementary Fig. S7A–S7C). Interestingly, there is also some overlap between seclidemstat and LSD1-downregulated and EWSR1::FLI1–downregulated genes, as well as seclidemstat and LSD1-downregulated and EWSR1::FLI1–upregulated genes, a pattern not seen with SP-2509 (Supplementary Fig. S7). The association between seclidemstat treatment and reversal of LSD1 function is stronger (Supplementary Fig. S7C) than for EWSR1::FLI1. Using GSEA, we found that LSD1-upregulated genes are downregulated by seclidemstat (NES, −2.4373; Fig. 2F) and LSD1-downregulated genes are upregulated by seclidemstat (NES, 2.16; Fig. 2G). For EWSR1::FLI1, activated and repressed genes were both more strongly associated with seclidemstat upregulation (NES, 1.8047 and NES, 1.3087, respectively; Supplementary Fig. S7D and S7E). There are several factors that may contribute to these findings. First is the reduced cell permeability expected with seclidemstat as compared with SP-2509, and second is the relatively lower dose used for seclidemstat (∼1 −mol/L) as compared with SP-2509 (2 μmol/L; ref. 32). Both may reduce the effective dose of seclidemstat in the same tissue culture conditions, and this is supported by the overall lower number of seclidemstat-regulated genes as compared with SP-2509 (Fig. 2A and B). Taken together, treatment with seclidemstat significantly affects both EWSR1::FLI1–regulated and LSD1-regulated genes and closely resembles SP-2509 treatment.

### Treatment with seclidemstat broadly alters gene regulation in FET-rearranged sarcomas

**Figure 3:**
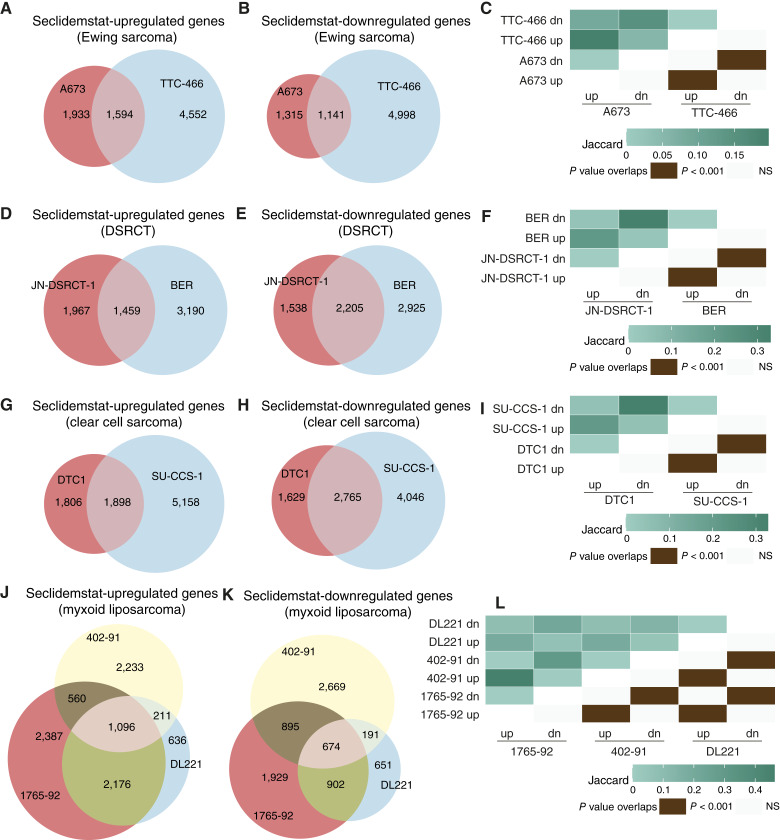
Seclidemstat alters the expression of thousands of genes in FET-rearranged sarcoma cell lines. **A–C,** Venn overlap analysis of seclidemstat (**A**) upregulated and (**B**) downregulated genes in A673 and TTC-466 Ewing sarcoma cell lines with the Jaccard index and *P* values of overlap (numbers inside each cell) shown in **C**. **D–F,** Venn overlap analysis of seclidemstat (**D**) upregulated and (**E**) downregulated genes in JN-DSRCT-1 and BER DSRCT cell lines with the Jaccard index and *P* values of overlap (numbers inside each cell) shown in **F**. **G–I,** Venn overlap analysis of seclidemstat (**G**) upregulated and (**H**) downregulated genes in DTC1 and SU-CCS-1 clear cell sarcoma cell lines with the Jaccard index and *P* values of overlap (numbers inside each cell) shown in **I**. **J–L,** Venn overlap analysis of seclidemstat (**J**) upregulated and (**K**) downregulated genes in 1765-92, 402-91, and DL221 myxoid liposarcoma cell lines with the Jaccard index and *P* values of overlap shown in **L**. dn, downregulated; NS, not significant; up, upregulated.

Given the activity observed in cell viability assays, we next asked whether seclidemstat also altered gene regulation in cell lines with other FET fusion proteins. As seen for A673 cells, seclidemstat treatment changed the expression of thousands of genes in all the tested cell lines (Fig. 3A–L). This was true for both upregulated genes (Fig. 3A, D, G, and J) and downregulated genes (Fig. 3B, E, H, and K). Additionally, there was significant functional overlap (i.e., common upregulated DEGs and common downregulated DEGs) in the genes regulated by seclidemstat across cell lines within each disease category (Fig. 3C, F, I, and L).

**Figure 4:**
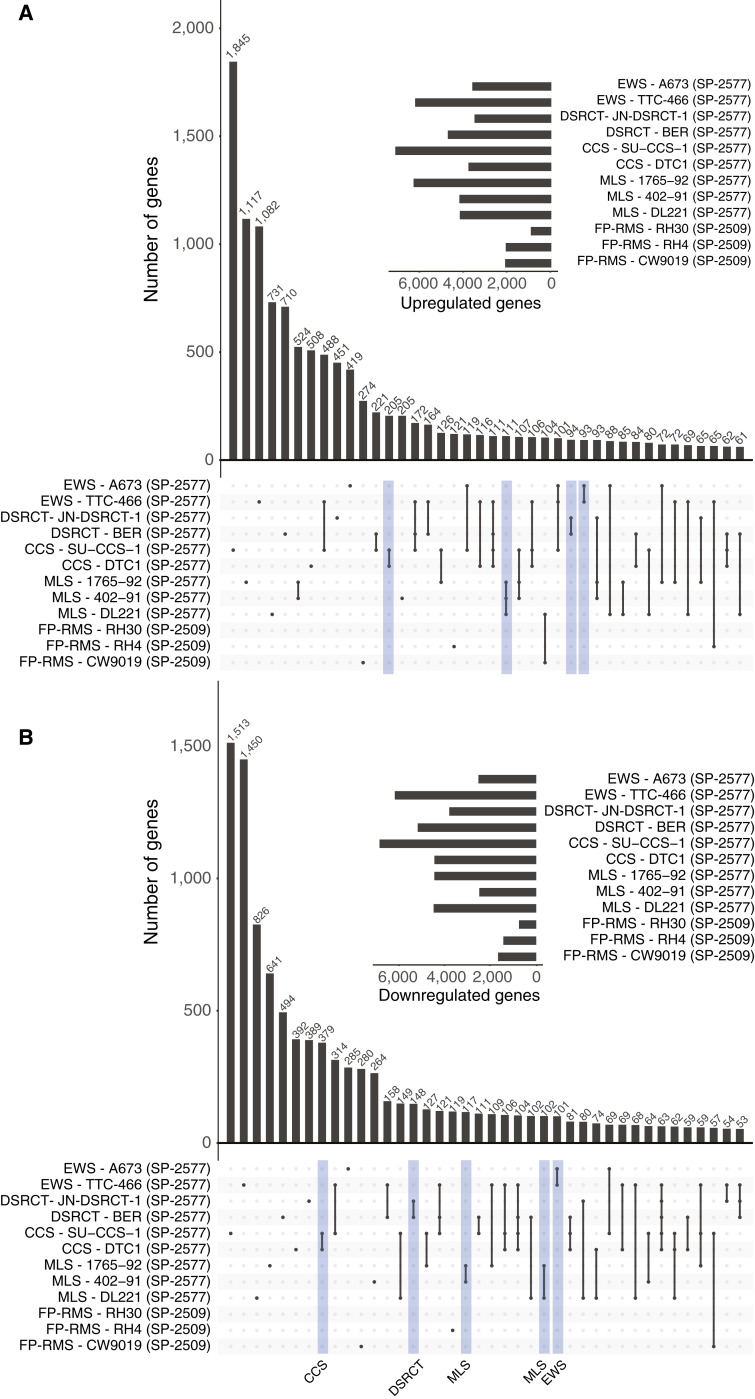
Seclidemstat regulates unique gene sets in each cell line and common biological pathways across cell lines. **A** and **B,** Overlap analysis via upset plots of seclidemstat (**A**) upregulated and (**B**) downregulated genes in all nine cell lines tested. Inset shows the total number of DEGs detected in each cell line. Blue boxes highlight the disease-specific gene sets. CCS, clear cell sarcoma; EWS, Ewing sarcoma; MLS, myxoid liposarcoma.

Though there was significant similarity in genes regulated by seclidemstat within a given disease, we also wanted to understand the genes and pathways commonly regulated by seclidemstat across all tested cell lines. We first used an upset plot to look at commonly regulated genes (Fig. 4A and B). These analyses revealed that, for both seclidemstat-upregulated and -downregulated genes, the predominant DEGs were unique to each cell line (Fig. 4A and B). There were also DEGs that were unique to each disease type, but these were typically far less abundant than DEGs unique to each cell line (Fig. 4A and B). There were so few commonly regulated DEGs that the category was not included in the top 40 categories in the upset plot (Fig. 4A and B) for either upregulated or downregulated DEGs.

We next used pathway analysis to evaluate the relevant pathways regulated by seclidemstat treatment across cell lines. For this analysis, we tested pathway signatures from MSigDB Curated Gene Sets (Supplementary Fig. S8A), Gene Ontology Biological Processes (Supplementary Fig. S7B), and Gene Ontology Molecular Function (Supplementary Fig. S8C). Gene signatures related to cell cycle, RNA processing, mitochondria and metabolism, embryonic stem cell programs, DNA replication, and TF and cofactor binding were downregulated following treatment with seclidemstat (Supplementary Fig. S8A–S8C). In contrast, gene signatures related to cell signaling, development, EWSR1::FLI1, and PAX3::FOXO1 repressed targets, and the cellular response to starvation were all upregulated in seclidemstat-treated cells (Supplementary Fig. S8A–S8C). Considered together, these analyses suggest that, regardless of somewhat unique DEG sets in each cell line, seclidemstat treatment downregulates cellular pathways involved in oncogenesis, such as increased proliferation and a more dedifferentiated state, and upregulated pathways involved in cell signaling, differentiation, and development that are repressed by fusion oncogenes. These findings suggest that there may be common mechanisms of transcriptional regulation in FET-rearranged tumors that are disrupted by treatment with noncompetitive LSD1 inhibitors.

To further explore whether the transcriptional activity of this class of compounds was specific to cells expression FET-rearranged oncogenes, we included additional transcriptional data (RNA-seq) from FP-RMS cells following treatment with 2 μmol/L SP-2509 for 48 hours. The FP-RMS samples clustered together in our UMAP analysis of all cell lines (Supplementary Fig. S6). As seen in other cell lines, treatment resulted in significant numbers of DEGs both upregulated and downregulated (Supplementary Fig. S9A and S9B). We found more variability in the intradisease overlap of DEGs between FP-RMS cell lines than for other FET fusions. Interestingly, overlap analysis with upset plots for all cell lines showed relatively little overlap between DEGs found in FP-RMS and those found in FET-rearranged sarcomas (Fig. 4A and B). Furthermore, when we included FP-RMS samples in our pathway analysis, we found more variability in the pathways altered across cell lines (Supplementary Fig. S10A–S10C) than when we focused only on cell lines containing FET rearrangements (Supplementary Fig. S8A–S8C). Notably, genes upregulated in alveolar RMS were downregulated by SP-2509 treatment in all FP-RMS cell lines (Supplementary Fig. S10A) but none of the FET-rearranged lines. Genes associated with hypoxia were downregulated (Supplementary Fig. S10A), as were genes associated with replication (Supplementary Fig. S10B) and genes encoding proteins that bind to DNA and cytoskeletal elements (Supplementary Fig. S10C). Genes related to autophagy were downregulated, and genes involved in development were both upregulated and downregulated depending on the cell line and specific developmental pathway (Supplementary Fig. S10B). Together, these data suggest both that treatment with *N’*-(2-hydroxybenzilidene)hydrazide regulates some common pathways across the sarcoma cell types tested in this study and that response of FET-rearranged sarcomas encompasses a distinct set of common pathways.

### FET fusion proteins show significant similarities in gene regulation

To evaluate the impact of seclidemstat treatment on the transcriptional function of FET fusion oncogenes, we first reanalyzed publicly available RNA-seq datasets for four cell lines with different FET fusions: A673 (as described above; bioRxiv 2024.01.31.578127), JN-DSRCT-1 (11), BER (11), and DTC1 (6). These datasets were downloaded and processed using our in-house RNA-seq pipeline to define the genes regulated by expression of the fusion. We also generated a new RNA-seq dataset (GSE267611) for EWSR1::ERG in TTC-466 cells using a similar approach as that described above for A673s. Briefly, we performed a standard KD–rescue experiment using a 3′ untranslated region–targeted shRNA to deplete endogenous EWSR1::ERG and rescued with a shRNA-resistant cDNA encoding EWSR1::ERG, similar to that reported for EWSR1::FLI1 in prior experiments (30, 31). TTC-466 cells tolerated EWSR1::ERG KD/rescue with expression of the rescue construct at similar expression levels seen for both EWSR1::FLI1 and EWSR1:ERG (Supplementary Fig. S11A), rescue of growth in soft agar comparable with that seen for EWSR1::FLI1 (Supplementary Fig. S11B and S11C), and rescue of an oncogenic transcriptional signature (Supplementary Fig. S11D). Having shown this KD/Rescue worked we then defined the EWSR1::ERG signature as genes differentially expressed in cells expressing the EWSR1:ERG rescue construct compared with the KD condition.

**Figure 5:**
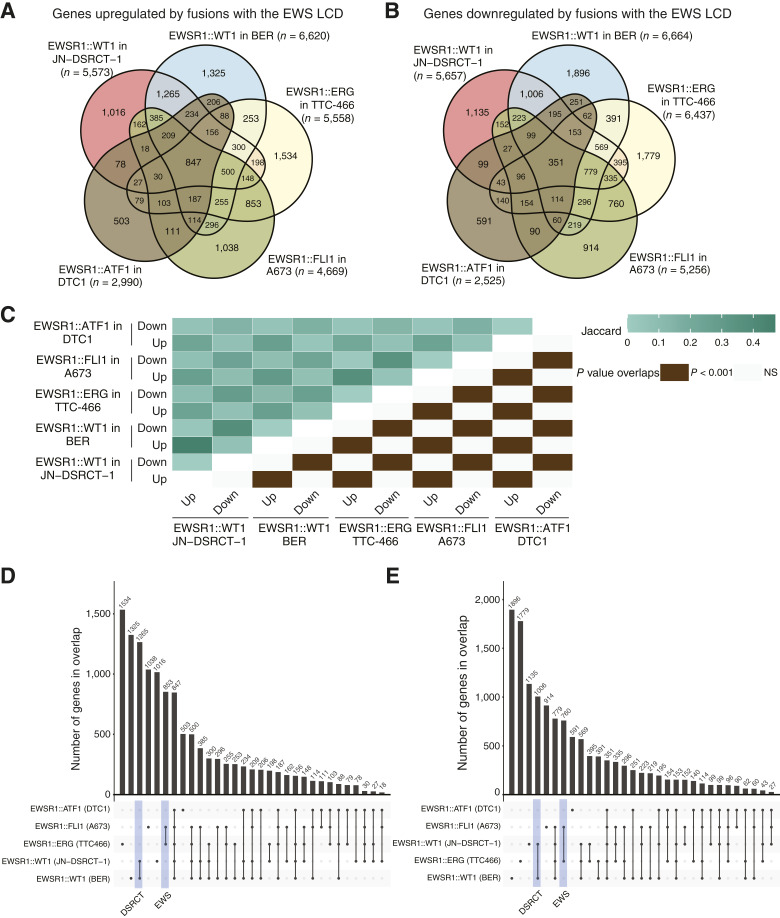
Different FET fusion proteins regulate common gene sets. **A–C,** Venn overlap analysis of FET fusion (**A**) activated and (**B**) repressed genes in A673, TTC-466, JN-DSRCT-1, BER, and DTC1 cell lines with the Jaccard indices for pairwise comparisons and *P* values of overlap shown in **C**. **D** and **E,** Overlap analysis via upset plots of FET fusion (**D**) activated and (**E**) repressed genes in all five cell lines tested. Down, downregulated; EWS, Ewing sarcoma; NS, not significant; Up, upregulated.

Overlap analyses of all five fusion transcriptional signatures revealed a large degree of overlap with 847 genes commonly upregulated and 351 genes commonly downregulated (Fig. 5A and B) and statistically significant overlaps in each pairwise comparison between the respective upregulated and downregulated genes from each fusion (Fig. 5C). These data confirm a prior report from Gedminas and colleagues (11) that there is significant overlap in the DEG signatures for EWSR1::WT1 in both JN-DSRCT-1 and BER, as well as between both DSRCT cell lines and the EWSR1::FLI1 signature (Fig. 4C). Notably, the EWSR1::ATF1 DEG signature also showed significant overlap with all other fusions tested (Fig. 5C). Despite these significant overlaps, the most prevalent DEG sets were unique to each cell line (Fig. 5D and E). The next most prevalent DEG sets were those common to each disease and then those common to all fusions for both upregulated and downregulated genes (Fig. 5D and E). There were more commonly upregulated genes than downregulated genes,, and this may reflect common mechanisms of transcriptional activation in the presence of these fusions.

### Seclidemstat treatment reverses FET fusion transcriptional activity

**Figure 6:**
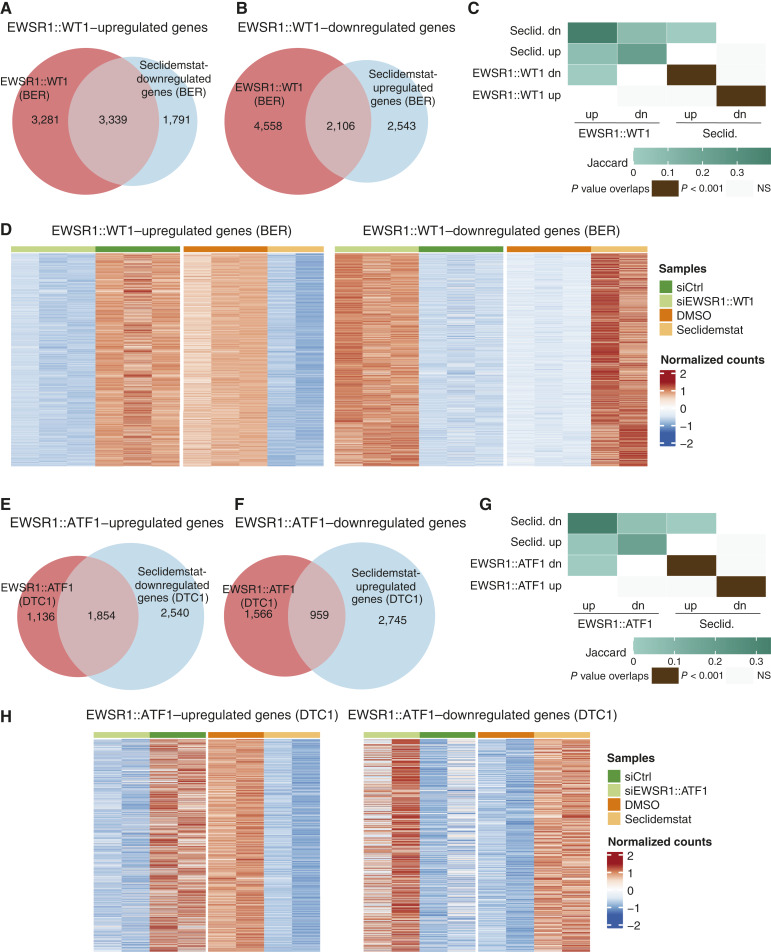
Seclidemstat reverses the transcriptional signature of EWSR1::WT1 and EWSR1::ATF1. **A–C,** Venn overlap analysis of (**A**) seclidemstat-downregulated genes and EWSR1::WT1–activated genes and (**B**) seclidemstat-upregulated genes and EWSR1::WT1–repressed genes in BER cells with the Jaccard index and *P* values of overlap shown in **C**. **D,** Heatmap analysis showing the effect of seclidemstat treatment on the EWSR1::WT1 transcriptional signature. Each row represents a DEG (adjusted *P* < 0.05), and each column is a separate biological replicate. **E–G,** Venn overlap analysis of (**E**) seclidemstat-downregulated genes and EWSR1::ATF1–activated genes and (**F**) seclidemstat-upregulated genes and EWSR1::ATF1–repressed genes in DTC1 cells with the Jaccard index and *P* values of overlap shown in **G**. **H,** Heatmap analysis showing the effect of seclidemstat treatment on the EWSR1::ATF1 transcriptional signature. Each row represents a DEG (adjusted *P* < 0.05), and each column is a separate biological replicate. dn, downregulated; Seclid., seclidemstat; NS, not significant; up, upregulated.

In our last analysis, we asked whether seclidemstat treatment blocks the transcriptional activity of FET fusion proteins in other diseases. In both JN-DSRCT-1 and BER cells, we found that seclidemstat downregulated a significant number of EWSR1::WT1–activated genes and upregulated a significant number of EWSR1::WT1 downregulated genes (Fig. 6A–C; Supplementary Fig. S12A–S12C). Unlike the results for EWSR1::FLI1 discussed above, seclidemstat downregulation showed no significant overlap with EWSR1::WT1–repressed genes, and the same was true for seclidemstat upregulation of EWSR1::WT1–activated genes (Fig. 6C; Supplementary Fig. S12C). Heatmap analyses in Fig. 6D; Supplementary Fig. S12D likewise show a reversal of the EWSR1::WT1 transcriptional signature following seclidemstat treatment. Importantly, further analysis focusing on the direct targets of EWSR1::WT1 showed that seclidemstat treatment downregulated EWSR1::WT1–activated genes and upregulated EWSR1::WT1–repressed genes. The results were largely the same for EWSR1::ATF–regulated genes in DTC1 cells (Fig. 6E–H) and EWSR1::ERG–regulated genes in TTC-466 cells (Supplementary Fig. S12E–S12H). In these cell lines, seclidemstat upregulated genes that were repressed by the fusion and *vice versa*, there was no significant overlap between seclidemstat upregulation and fusion-activated genes or between seclidemstat downregulation and fusion-repressed genes, and reversal of fusion activity was seen at direct targets of the fusion (Supplementary Fig. S13A–S13C). These data indicate that seclidemstat reverses the transcriptional activity of multiple FET fusion proteins, specifically EWSR1::FLI1, EWSR1::ERG, EWSR1::WT1, and EWSR1::ATF1. This finding is largely consistent with the common set of pathways that change upon seclidemstat treatment, as shown in Supplementary Fig. S8, and the degree of similarity in the genes that are regulated by the different fusions in different diseases, as shown in Fig. 5.

## Discussion

Taken together, we found that seclidemstat shows potent activity against cells derived from patient tumors with multiple different FET and non-FET fusion oncogenes. Similar to prior reports in both Ewing sarcoma and DSRCT (32, 60), cell viability was affected by both of the noncompetitive inhibitors, SP-2509 and seclidemstat, but not OG-L002, indicating that for all of the tumor types tested, inhibition of the catalytic function of LSD1 is not a sufficient antitumor strategy. One possible explanation for this has been demonstrated by Sehrawat and colleagues (61), in which treatment with SP-2509, but no catalytic LSD1 inhibitor, disrupted interactions between LSD1 and a critical coregulator, ZNF217. LSD1 is a part of multiple chromatin coregulatory complexes and has been shown to have noncatalytic activity, including displacing histone acetyltransferases and functioning as a scaffold to interact with other proteins like HDAC, TFs, and ubiquitin-specific proteases (62–64). The specific composition of LSD1 complexes and their roles in Ewing sarcoma have not been clearly defined, and this is an area of continued study.

Another likely possibility is LSD1-independent activity of the compounds. Others have suggested that SP-2509 is a compound with pan-assay interference inhibition (65–67) which may result in off-target cytotoxic effects. Notably SP-2513 contains the same hydrazide core, with a 2-chloro substituted for the 2-hydroxy of SP-2509, and this compound shows no activity in cell-based assays. Alternatively, there may still be off-target effects related to the *N’*-(2-hydroxybenzilidene)hydrazide core that lead to reversal of FET fusion protein activity. This is an active area of investigation, and our early results suggest that the *N’*-(2-hydroxybenzilidene)hydrazide core can block the transcriptional activity of EWSR1::FLI1, but the biological mechanisms by which this occurs are poorly defined (bioRxiv 2025.06.20.660795). With these data in mind, we think it is likely that the anti–FET fusion activity seen in this study is likely LSD1-independent. More studies are needed to understand the mechanisms that lead to transcriptional changes after treatment, as these are likely secondary or tertiary effects downstream from the direct targets of SP-2509 and seclidemstat.

This is the largest study to date comparing the transcriptional activity of different FET fusion proteins analyzed together. The size of the dataset, including biological replicates and untreated controls, combined with systematic sample preparation and sequencing protocols followed to minimize batch effect make this particularly useful as a resource for the study of this family of cancers. These data will allow exploration of shared and distinct mechanisms underlying the oncogenesis of these cell lines. We were interested to see both a significant portion of fusion-mediated gene regulation unique to each cell line tested as well as a reasonably large number of commonly regulated genes across the five cell lines tested. This was particularly true for fusion-activated genes, with 847 commonly upregulated gene targets. Whereas the fusions from each disease have different TF DBDs in the C-terminal portion of the protein, they may nonetheless use common transcriptional regulatory mechanisms. For example, the Ewing sarcoma LCD is thought to mediate a number of its gene regulatory functions (10, 25, 68, 69), a new genomic approach to probe the regions of the genome that interact with the Ewing sarcoma LCD has revealed other TFs possibly enriched with fusion proteins like EWSR1::FLI1 (70). This may prove to be true for other FET fusion proteins. There were fewer common FET fusion–repressed genes. The overlap may reflect commonalities in cells of origins, whereas the differences may reflect repression of developmental and signaling pathways unique to each cell of origin for each type of disease and within each patient.

In line with our prior findings for Ewing sarcoma (18), treatment with noncompetitive LSD1 inhibitors reverses the global transcriptional activity of FET fusion proteins, at both direct and indirect target genes. A significant number of genes modulated by treatment with seclidemstat were unique to each cell line, and this is consistent with uniquely regulated genes being the most prevalent gene class when comparing all fusions. However, pathway analysis showed significant functional overlap in the pathways regulated by seclidemstat treatment in the FET-rearranged cell lines. Notably, signatures related to proliferation (cell cycle and replication), RNA processing, and stem cell pathways were downregulated by seclidemstat. Cell signaling and various developmental gene classes were upregulated by seclidemstat. Taken together, these results comprise an orthogonal analytic approach supporting the finding that seclidemstat effectively reverses the oncogenic phenotypes induced by FET fusion proteins.

Seclidemstat thus showed comparable pharmacodynamic activity with SP-2509 in these preclinical assay systems, with significant disruption of FET fusion protein function and LSD1. Seclidemstat is currently in clinical trials for patients with Ewing sarcoma and related tumors, as well as myelodysplastic syndrome and chronic myelomonocytic leukemia, and additional prospective, randomized controlled studies are needed to confirm activity in these patients. Based on our findings that seclidemstat treatment interferes with the transcriptional function of other fusion proteins, seclidemstat treatment may also be beneficial in other FET-rearranged sarcomas. Additional pharmacokinetic and pharmacodynamic studies are needed to understand how seclidemstat treatment leads to transcriptional changes at 48 hours, whether the current seclidemstat dosing strategy used in the clinic affects FET fusion protein activity in patients, and how this might be leveraged in the context of other treatment modalities. SP-2509 has been shown to synergize with HDAC inhibitors in Ewing sarcoma (71), and this may be an interesting combination to explore. Additionally, others have found that the process of tumors becoming resistant to chemotherapy confers collateral sensitivity to SP-2509 (72) such that seclidemstat could be incorporated into an evolutionary treatment framework for sarcoma (73). Seclidemstat thus remains a promising new treatment strategy for patients with FET-rearranged sarcomas, and future studies are required to understand how to best use this compound in the clinic.

## Supplementary Material

Supplementary Table S1 Supplementary Table S1. Compiled IC50s for SP-2509, SP-2513, and OG-L002 for fusion-positive rhabdomyosarcoma cell lines and replicates.

Supplementary Figure S1 Figure S1. Additional Ewing sarcoma replicate dose response curves for seclidemstat

Supplementary Figure S2 Figure S2. Additional DSRCT, clear cell sarcoma, and myxoid liposarcoma replicate dose response curves for seclidemstat

Supplementary Figure S3 Figure S3. Fusion-positive rhabdomyosarcoma replicate dose response curves for SP-2509.

Supplementary Figure S4 Figure S4. Visualization of the fusion calls from the EnFusion pipeline analysis of RNA-seq data show (A) EWSR1::FLI1 in A673 cells, (B) EWSR1::ERG in TTC-466 (C) JN-DSRCT-1 and (D) BER cells, EWSR1::ATF1 in (E) SU-CCS-1 and (F) DTC1 cells, and FUS::DDIT3 in (G) 1765-92, (H) 402-91, and (I) DL221 cells

Supplementary Figure S5 Figure S5. Heatmap and hierarchical clustering of the transcript levels of known LSD1 interactors across cell lines included in this study.

Supplementary Figure S6 Figure S6. UMAP plot showing clustering of all analyzed RNA-seq samples.

Supplementary Figure S7 Figure S7. (A-C) Venn overlap analysis of (A) EWSR1::FLI1 activated, LSD1 activated, SP2509 downregulated, and seclidemstat downregulated genes; and (B) EWSR1::FLI1 repressed, LSD1 repressed, SP-2509 upregulated, and seclidemstat upregulated genes in A673 cells with the Jaccard index and p-values of overlap shown in (C). (D-E) Gene set enrichment analysis of (D) EWSR1::FLI1 activated and (E) EWSR1::FLI1 repressed genes with seclidemstat gene regulation as the rank-ordered list. Normalized enrichment score (NES), p-value, and multiple hypothesis adjusted p-values are shown in inset tables.

Supplementary Figure S8 Figure S8. (A-C) Pathway analysis for seclidemstat regulated genes in 9 FET-rearranged cell lines visualized with a dot plot using (A) MSigDB curated gene sets (B) gene ontology biological process, and (C) gene ontology molecular function gene signatures.

Supplementary Figure S9 Figure S9. Venn overlap analysis of SP-2509 (A) up- and (B) downregulated genes in fusion-positive rhabdomyosarcoma cell lines with the Jaccard index and p-values of overlap shown in (C).

Supplementary Figure S10 Figure S10. Pathway analysis for N’-(2-hydroxybenzilidene)hydrazide regulated genes in all tested cell lines visualized with a dot plot using (A) MSigDB curated gene sets (B) gene ontology biological process, and (C) gene ontology molecular function gene signatures.

Supplementary Figure S11 Figure S11. Principal component 2 on the y-axis is plotted against principal component 1 on the x-axis. Different cell conditions are depicted in different colors and different replicates are represented with different shapes.

Supplementary Figure S12 Figure S12. Venn overlap analysis of (A) seclidemstat downregulated genes and EWSR1::WT1 activated genes and (B) seclidemstat upregulated genes and EWSR1::WT1 repressed genes in JN-DSRCT-1 cells with the Jaccard index and p-values of overlap shown in (C). (D) Heatmap analysis showing the effect of seclidemstat treatment on the EWSR1::WT1 transcriptional signature. Each row represents a differentially expressed gene (adjusted p < 0.05) and each column is a separate biological replicate. (E-G) Venn overlap analysis of (E) seclidemstat downregulated genes and EWSR1::ERG activated genes and (F) seclidemstat upregulated genes and EWSR1::ERG repressed genes in TTC-466 cells with the Jaccard index and p-values of overlap shown in (G). (H) Heatmap analysis showing the effect of seclidemstat treatment on the EWSR1::ERG1 transcriptional signature. Each row represents a differentially expressed gene (adjusted p < 0.05) and each column is a separate biological replicate.

Supplementary Figure S13 Figure S13. Matrices showing GSEA results for various treatments on direct targets of (A) EWSR1::FLI1, (B) EWSR1::ATF1, and (C) EWSR1::WT1. Direct target gene sets were previously published (Refs. 6, 50, and 51). Briefly direct targets were defined as those genes 1) near a fusion protein bound locus or fusion-mediated chromatin loop and 2) with differential expression upon genetic depletion of the fusion. The coloring of each box represents the p-value for that GSEA analysis and the normalized enrichment score is reported inset in each box.
