# Supplementary material for: Seclidemstat (SP-2577) Induces Transcriptomic Reprogramming and Cytotoxicity in Multiple Fusion–Positive Sarcomas
Source: Cancer Res Commun. 2025 Sep 10;5(9):1584–98. doi: 10.1158/2767-9764.CRC-24-0296 (PMC12421227; doi:10.1158/2767-9764.CRC-24-0296)
Supplement: Supplementary Figure S1 — Figure S1. Additional Ewing sarcoma replicate dose response curves for seclidemstat [file crc-24-0296_supplementary_figure_s1_suppsf1.pdf]

Supplementary Figure 1

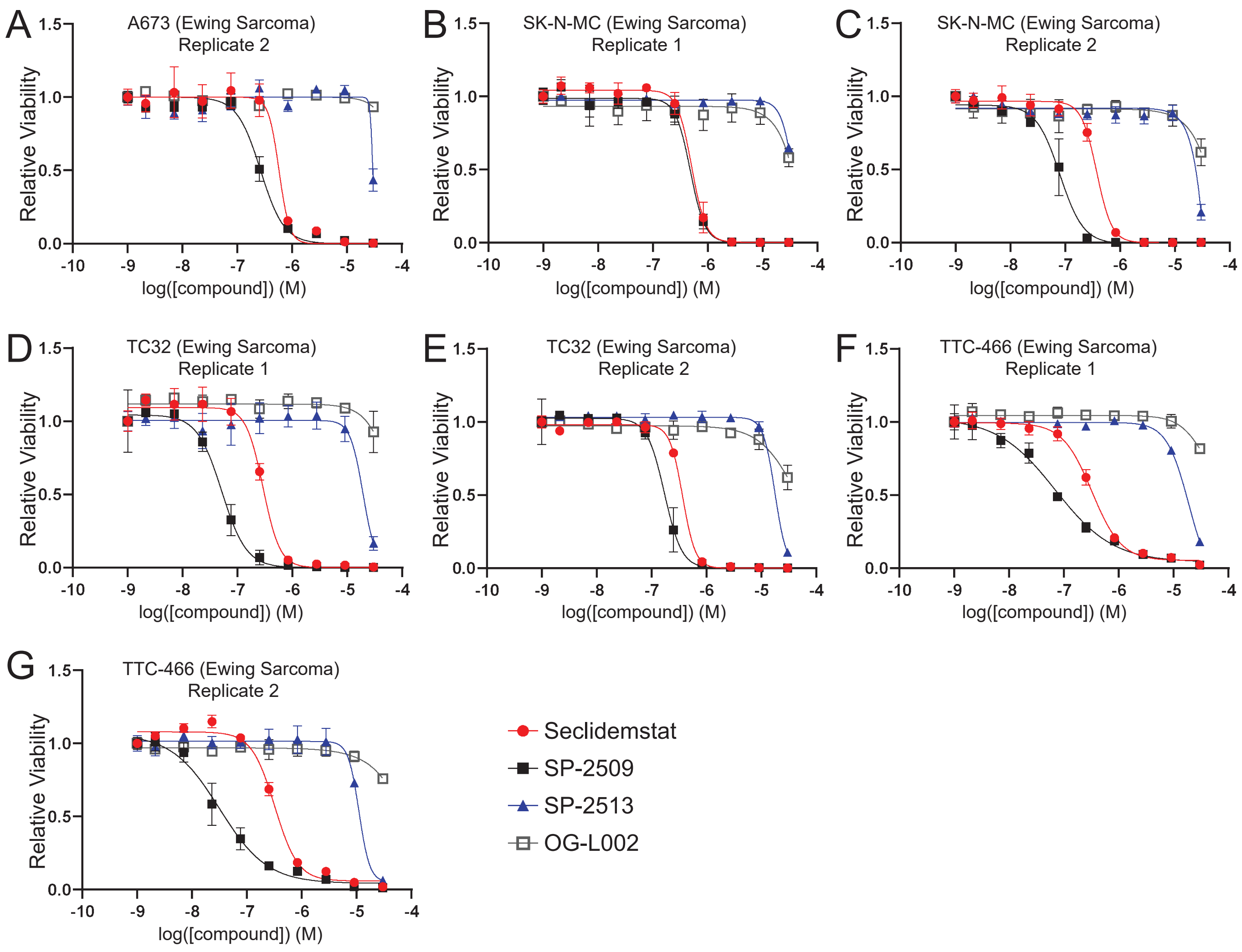

Supplementary Figure 1. (A-G) Additional Ewing sarcoma replicate dose response curves for seclidemstat (red/circle), SP-2509 (black/closed square), SP-2513 (blue/triangle), and OG-L002 (gray/open square) in (A) A673, (B,C) SK-N-MC, (D,E) TC32, and (F,G) TTC-466 cells. Each graph displays data for a single biological replicate. Mean values of 3 technical replicates are shown with standard deviation. Calculated curves of best fit are also shown.
