# Supplementary material for: Seclidemstat (SP-2577) Induces Transcriptomic Reprogramming and Cytotoxicity in Multiple Fusion–Positive Sarcomas
Source: Cancer Res Commun. 2025 Sep 10;5(9):1584–98. doi: 10.1158/2767-9764.CRC-24-0296 (PMC12421227; doi:10.1158/2767-9764.CRC-24-0296)
Supplement: Supplementary Figure S2 — Figure S2. Additional DSRCT, clear cell sarcoma, and myxoid liposarcoma replicate dose response curves for seclidemstat [file crc-24-0296_supplementary_figure_s2_suppsf2.pdf]

Supplementary Figure 2

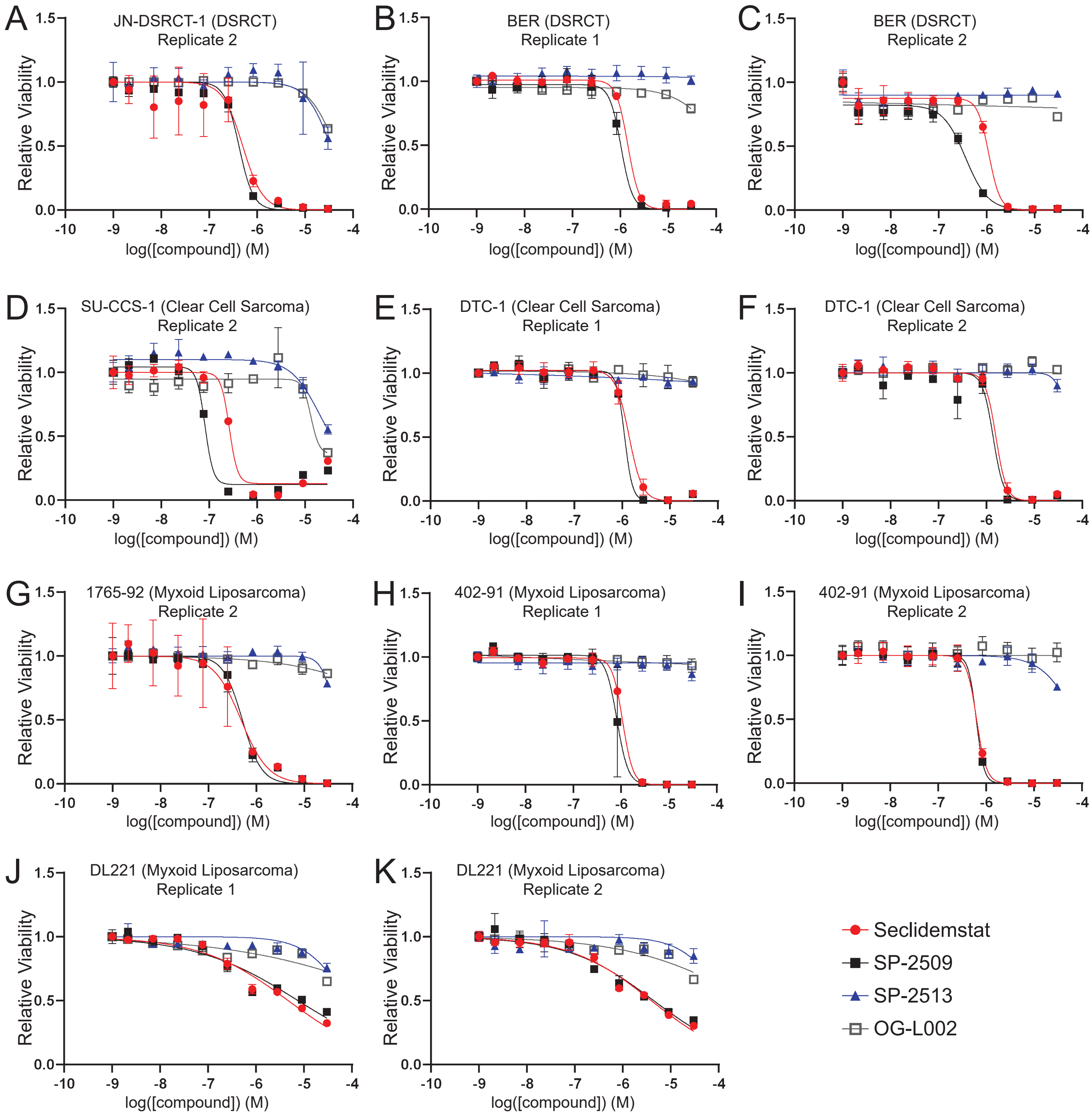

Supplementary Figure 2. (A-G) Additional DSRCT, clear cell sarcoma, and myxoid liposarcoma replicate dose response curves for seclidemstat (red/circle), SP-2509 (black/closed square), SP-2513 (blue/triangle), and OG-L002 (gray/open square) in (A) JN-DSRCT-1, (B,C) BER, (D) SU-CCS-1, (E,F) DTC1, (G) 1765-92, (H,I) 402-91, and (J,K) DL221 cells. Each graph displays data for a single biological replicate. Mean values of 3 technical replicates are shown with standard deviation. Calculated curves of best fit are also shown.
