# Supplementary material for: Seclidemstat (SP-2577) Induces Transcriptomic Reprogramming and Cytotoxicity in Multiple Fusion–Positive Sarcomas
Source: Cancer Res Commun. 2025 Sep 10;5(9):1584–98. doi: 10.1158/2767-9764.CRC-24-0296 (PMC12421227; doi:10.1158/2767-9764.CRC-24-0296)
Supplement: Supplementary Figure S3 — Figure S3. Fusion-positive rhabdomyosarcoma replicate dose response curves for SP-2509. [file crc-24-0296_supplementary_figure_s3_suppsf3.pdf]

Supplementary Figure 3

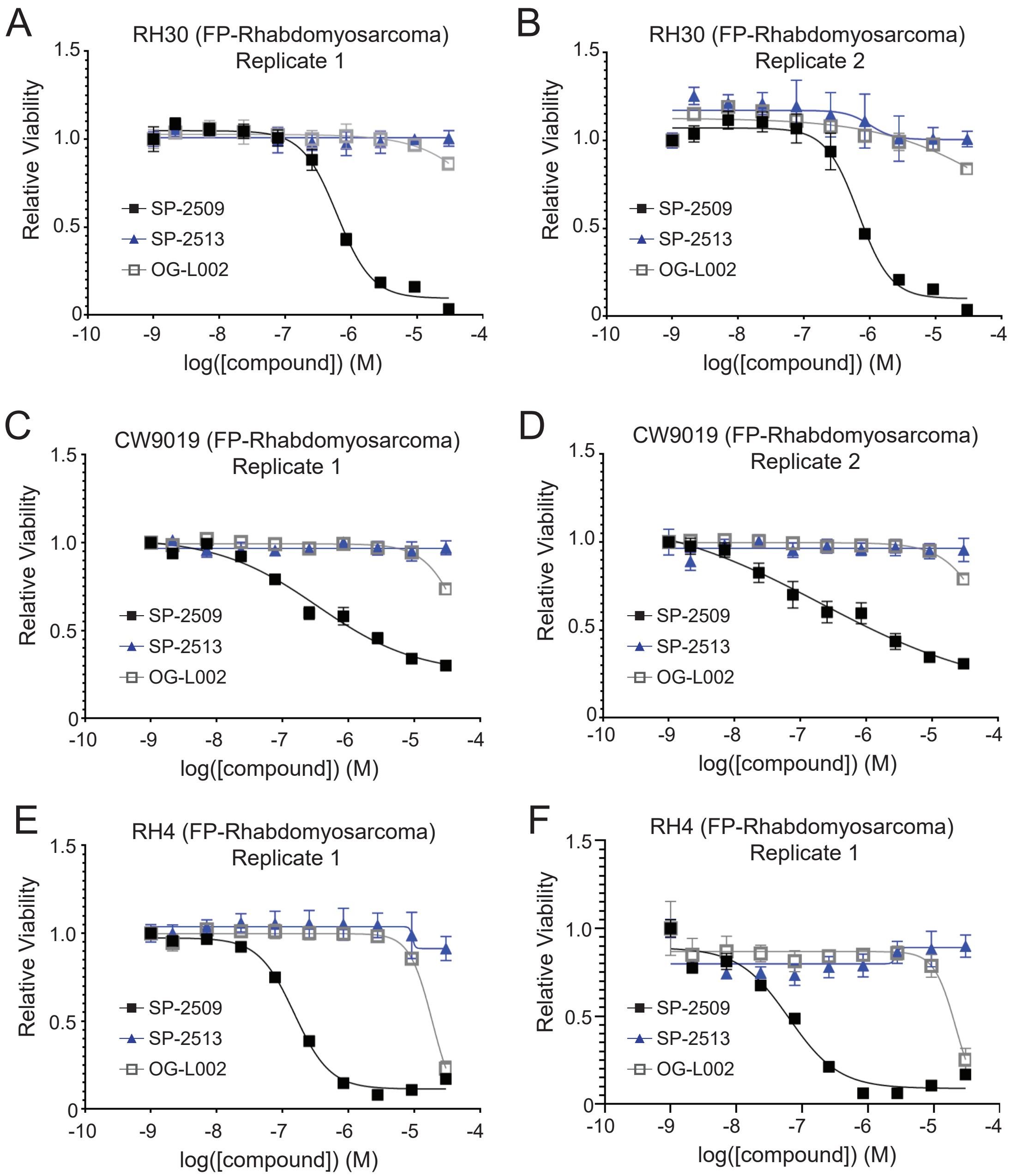

Supplementary Figure 3. (A-F) Fusion-positive rhabdomyosarcoma replicate dose response curves for SP-2509 (black/closed square), SP-2513 (blue/triangle), and OG-L002 (gray/open square) in (A,B) RH30, (C,D) CW9019, and (E,F) RH4. Each graph displays data for a single biological replicate. Mean values of 3 technical replicates are shown with standard deviation. Calculated curves of best fit are also shown.
