# Supplementary material for: Seclidemstat (SP-2577) Induces Transcriptomic Reprogramming and Cytotoxicity in Multiple Fusion–Positive Sarcomas
Source: Cancer Res Commun. 2025 Sep 10;5(9):1584–98. doi: 10.1158/2767-9764.CRC-24-0296 (PMC12421227; doi:10.1158/2767-9764.CRC-24-0296)
Supplement: Supplementary Figure S4 — Figure S4. Visualization of the fusion calls from the EnFusion pipeline analysis of RNA-seq data show (A) EWSR1::FLI1 in A673 cells, (B) EWSR1::ERG in TTC-466 (C) JN-DSRCT-1 and (D) BER cells, EWSR1::ATF1 in (E) SU-CCS-1 and (F) DTC1 cells, and FUS::DDIT3 in (G) 1765-92, (H) 402-91, and (I) DL221 cells [file crc-24-0296_supplementary_figure_s4_suppsf4.pdf]

Supplementary Figure 4

A

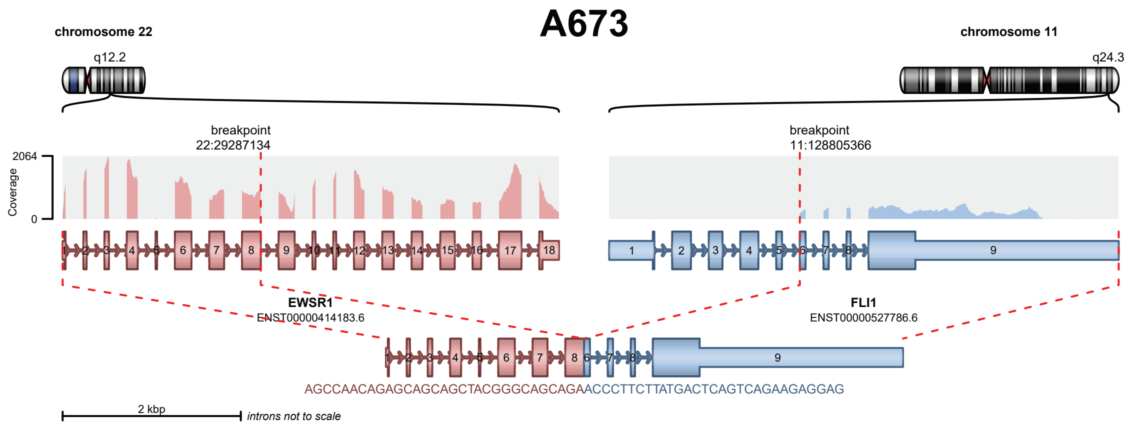

SUPPORTING READ COUNT

Split reads in EWSR1 = 8

Split reads in FLI1 = 64

Discordant mates = 3

B

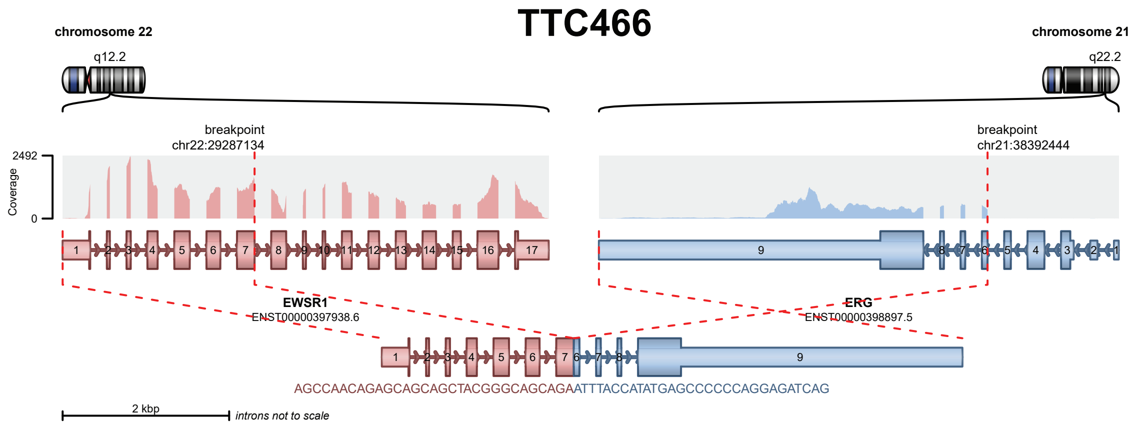

SUPPORTING READ COUNT

Split reads = 119

Discordant mates = 16

C

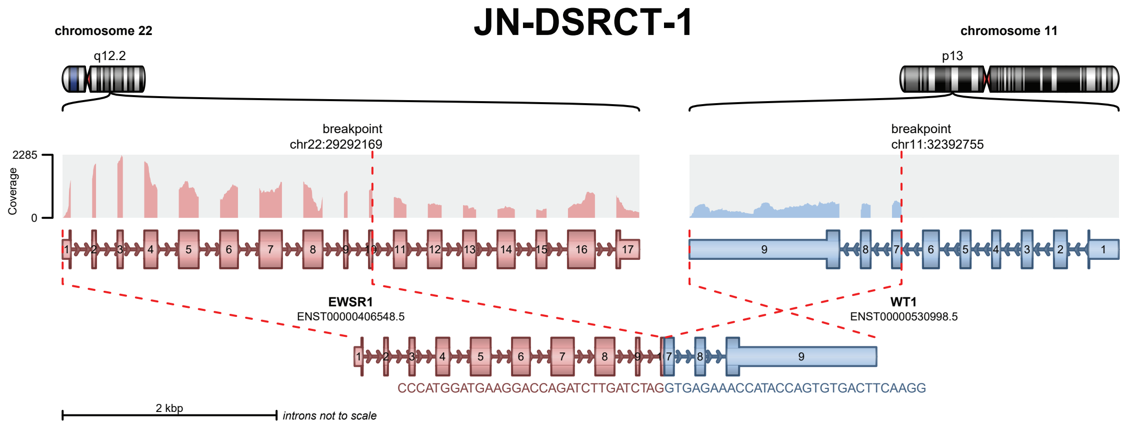

SUPPORTING READ COUNT

Split reads = 122

Discordant mates = 16

Supplementary Figure 4 (cont.)

SUPPORTING READ COUNT

Split reads = 196
Discordant mates = 27

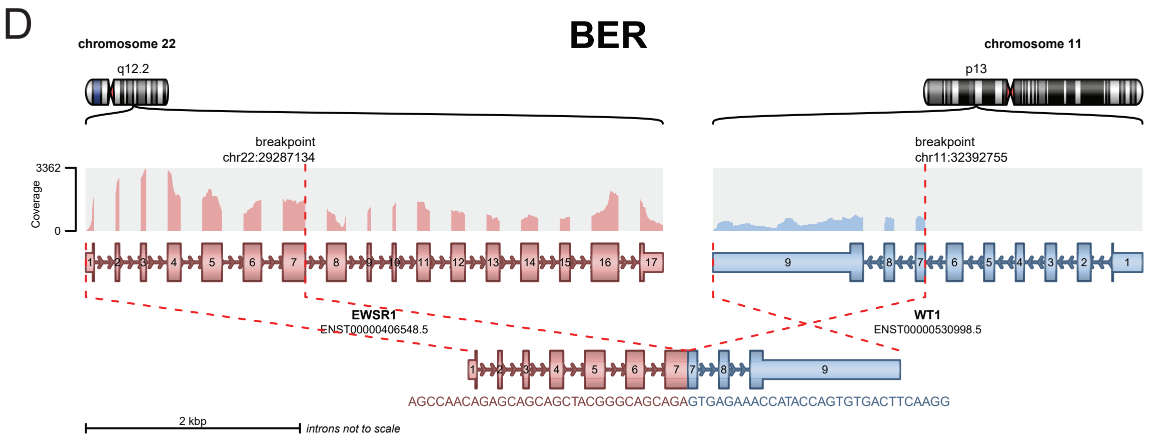

SUPPORTING READ COUNT

Split reads = 66
Discordant mates = 1

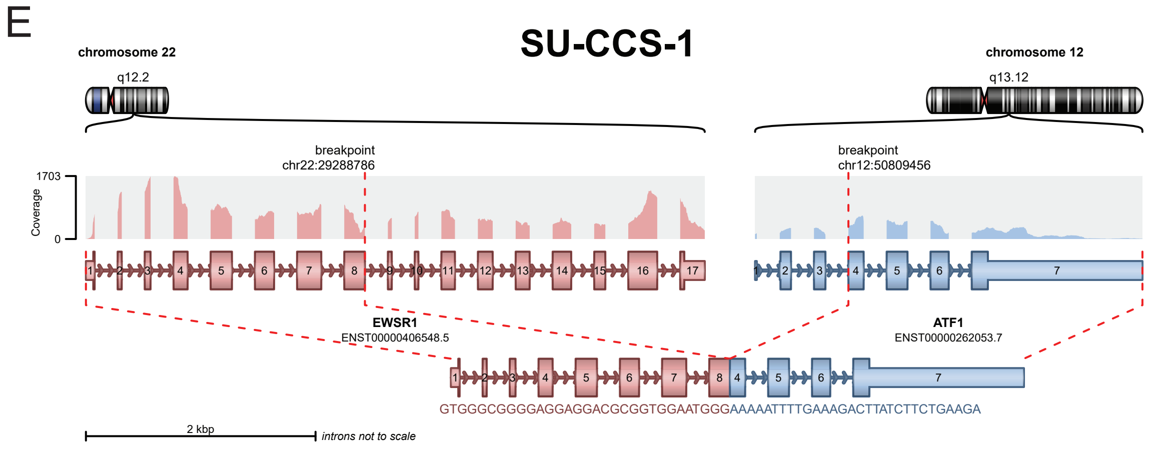

SUPPORTING READ COUNT

Split reads = 17
Discordant mates = 3

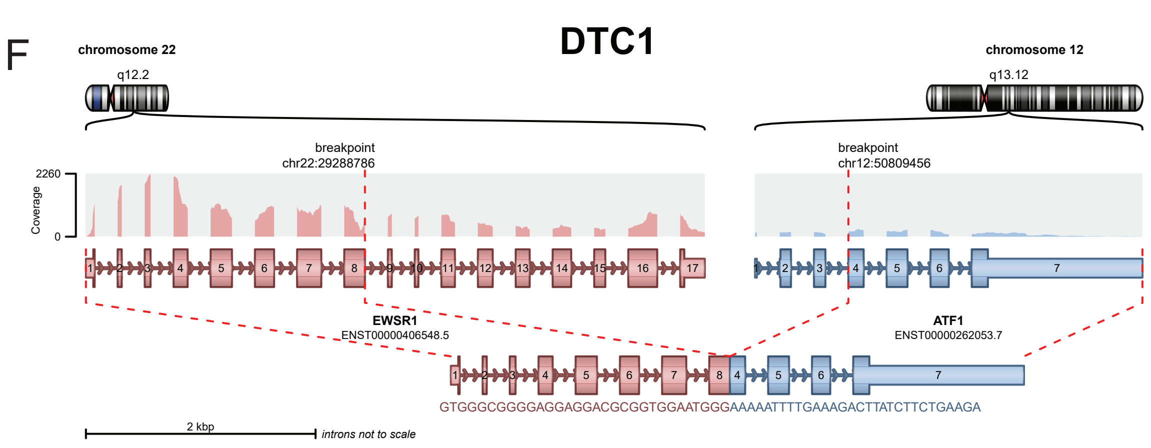

Supplementary Figure 4 (cont.)

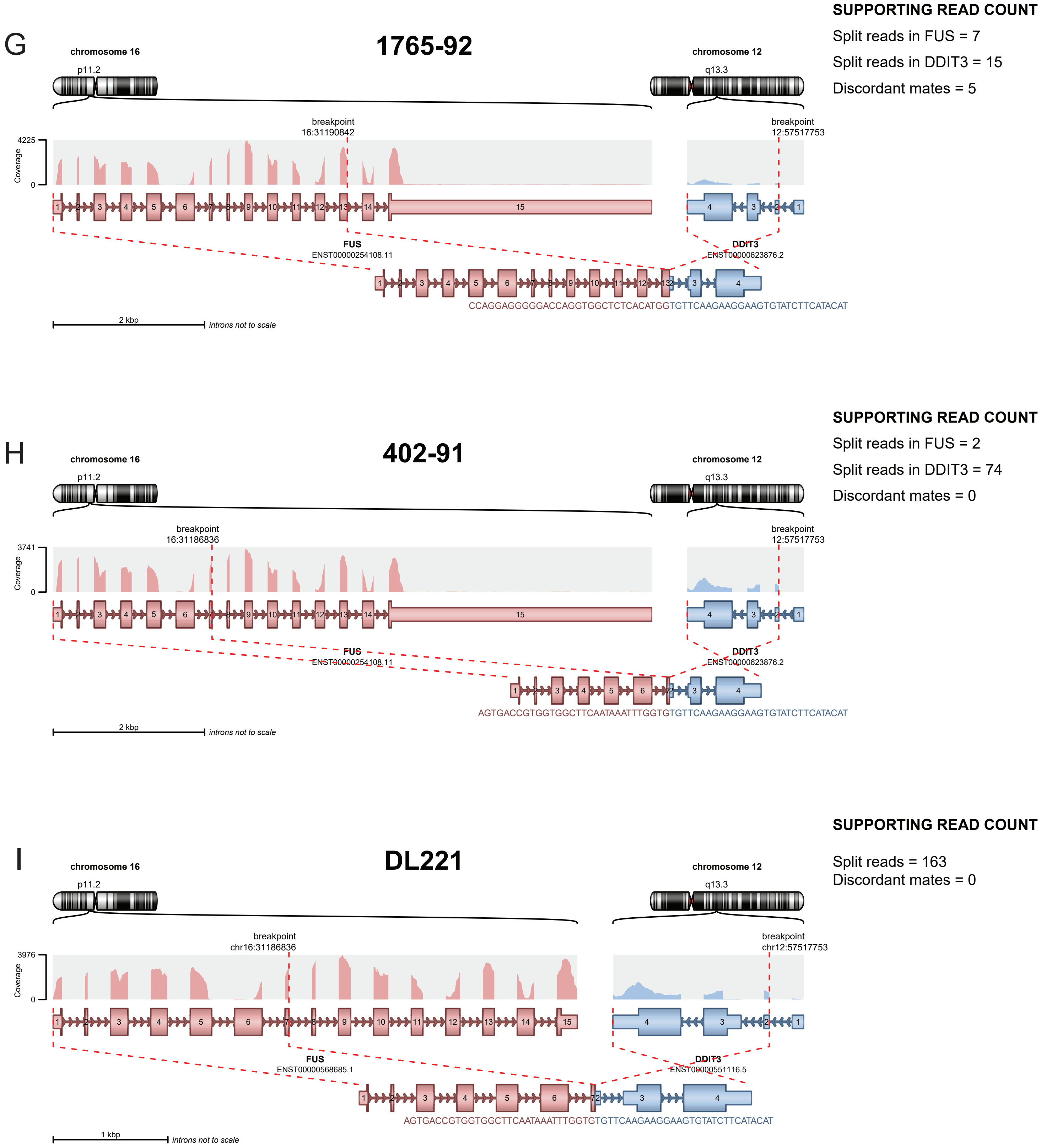

Supplementary Figure 4. (A-I) Visualization of the fusion calls from the EnFusion pipeline analysis of RNA-seq data show (A) *EWSR1::FLI1* in A673 cells, (B) *EWSR1::ERG* in TTC-466 cells, *EWSR1::WT1* in (C) JN-DSRCT-1 and (D) BER cells, *EWSR1::ATF1* in (E) SU-CCS-1 and (F) DTC1 cells, and *FUS::DDIT3* in (G) 1765-92, (H) 402-91, and (I) DL221 cells.
