# Supplementary material for: Seclidemstat (SP-2577) Induces Transcriptomic Reprogramming and Cytotoxicity in Multiple Fusion–Positive Sarcomas
Source: Cancer Res Commun. 2025 Sep 10;5(9):1584–98. doi: 10.1158/2767-9764.CRC-24-0296 (PMC12421227; doi:10.1158/2767-9764.CRC-24-0296)
Supplement: Supplementary Figure S5 — Figure S5. Heatmap and hierarchical clustering of the transcript levels of known LSD1 interactors across cell lines included in this study. [file crc-24-0296_supplementary_figure_s5_suppsf5.pdf]

Supplementary Figure 5

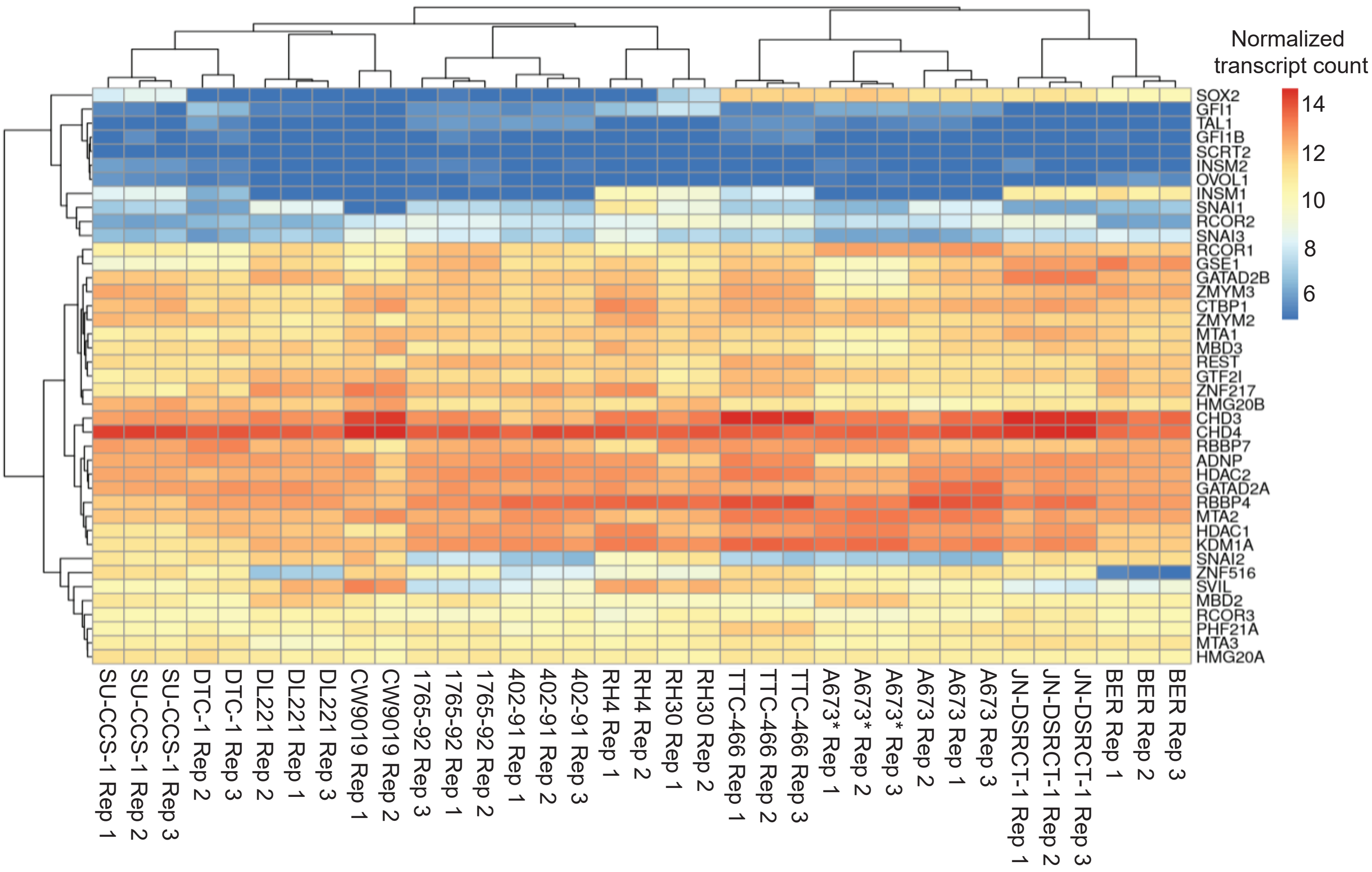

Supplementary Figure 5. Heatmap and hierarchical clustering of the transcript levels of known LSD1 interactors across cell lines included in this study. Transcript counts shown are the normalized values prior to differential expression analysis.
