# Supplementary material for: Seclidemstat (SP-2577) Induces Transcriptomic Reprogramming and Cytotoxicity in Multiple Fusion–Positive Sarcomas
Source: Cancer Res Commun. 2025 Sep 10;5(9):1584–98. doi: 10.1158/2767-9764.CRC-24-0296 (PMC12421227; doi:10.1158/2767-9764.CRC-24-0296)
Supplement: Supplementary Figure S6 — Figure S6. UMAP plot showing clustering of all analyzed RNA-seq samples. [file crc-24-0296_supplementary_figure_s6_suppsf6.pdf]

Supplementary Figure 6

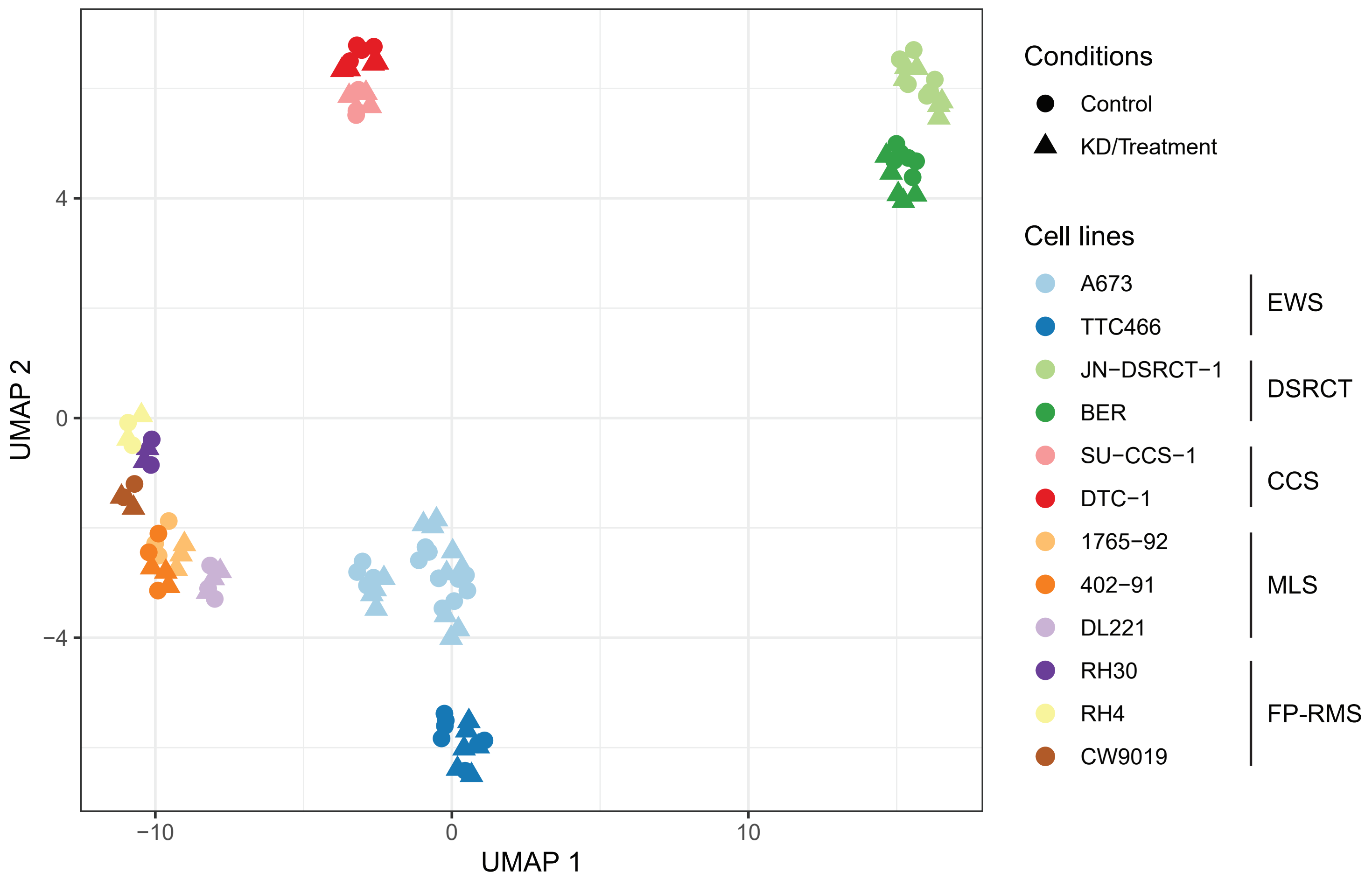

Supplementary Figure 6. UMAP plot showing clustering of all analyzed RNA-seq samples.
