# Supplementary material for: Seclidemstat (SP-2577) Induces Transcriptomic Reprogramming and Cytotoxicity in Multiple Fusion–Positive Sarcomas
Source: Cancer Res Commun. 2025 Sep 10;5(9):1584–98. doi: 10.1158/2767-9764.CRC-24-0296 (PMC12421227; doi:10.1158/2767-9764.CRC-24-0296)
Supplement: Supplementary Figure S7 — Figure S7. (A-C) Venn overlap analysis of (A) EWSR1::FLI1 activated, LSD1 activated, SP2509 downregulated, and seclidemstat downregulated genes; and (B) EWSR1::FLI1 repressed, LSD1 repressed, SP-2509 upregulated, and seclidemstat upregulated genes in A673 cells with the Jaccard index and p-values of overlap shown in (C). (D-E) Gene set enrichment analysis of (D) EWSR1::FLI1 activated and (E) EWSR1::FLI1 repressed genes with seclidemstat gene regulation as the rank-ordered list. Normalized enrichment score (NES), p-value, and multiple hypothesis adjusted p-values are shown in inset tables. [file crc-24-0296_supplementary_figure_s7_suppsf7.pdf]

Supplementary Figure 7

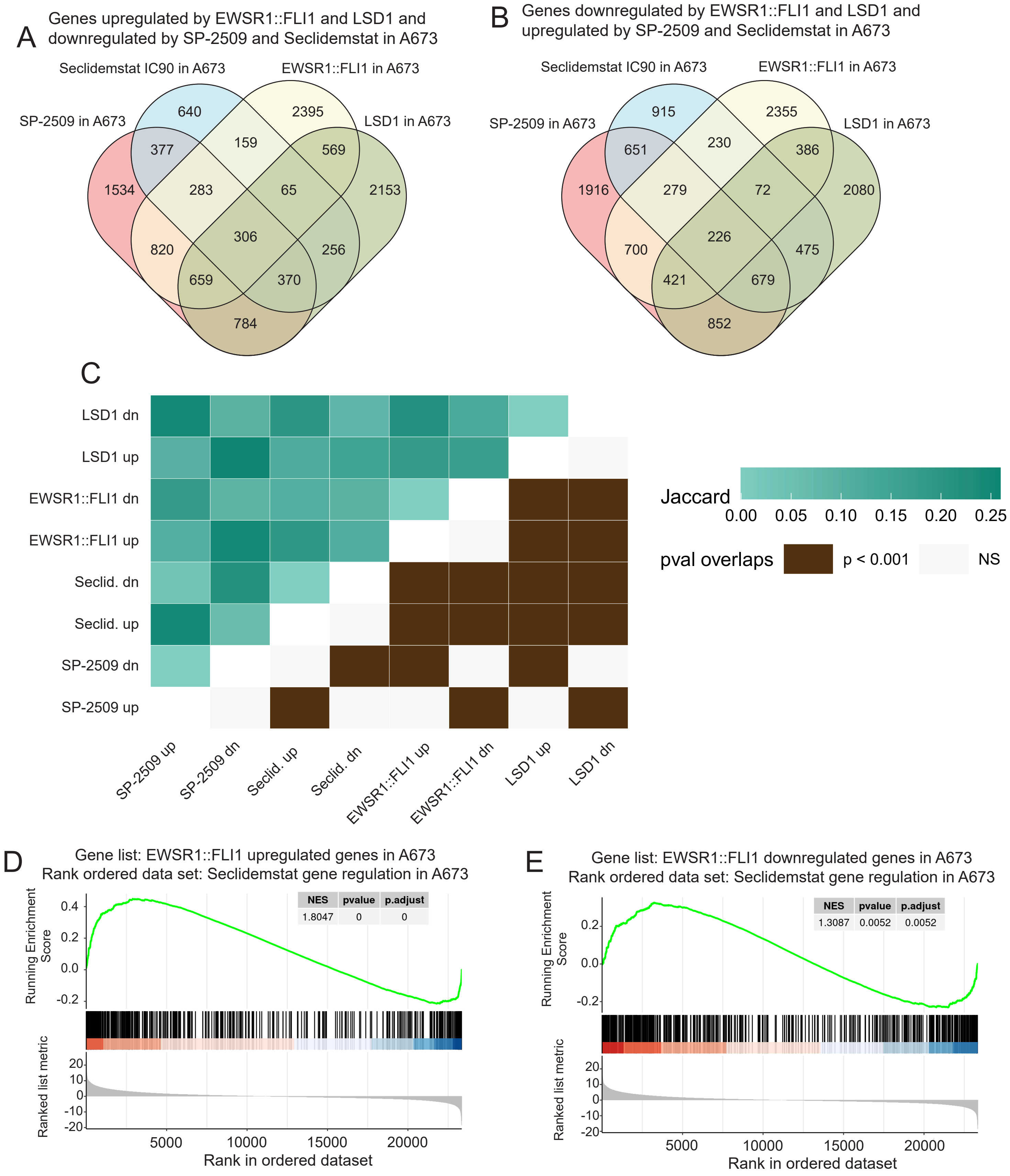

Supplementary Figure 7. (A-C) Venn overlap analysis of (A) EWSR1::FLI1 activated, LSD1 activated, SP-2509 downregulated, and seclidemstat downregulated genes; and (B) EWSR1::FLI1 repressed, LSD1 repressed, SP-2509 upregulated, and seclidemstat upregulated genes in A673 cells with the Jaccard index and p-values of overlap shown in (C). (D-E) Gene set enrichment analysis of (D) EWSR1::FLI1 activated and (E) EWSR1::FLI1 repressed genes with seclidemstat gene regulation as the rank-ordered list. Normalized enrichment score (NES), p-value, and multiple hypothesis adjusted p-values are shown in inset tables.
