# Supplementary material for: Seclidemstat (SP-2577) Induces Transcriptomic Reprogramming and Cytotoxicity in Multiple Fusion–Positive Sarcomas
Source: Cancer Res Commun. 2025 Sep 10;5(9):1584–98. doi: 10.1158/2767-9764.CRC-24-0296 (PMC12421227; doi:10.1158/2767-9764.CRC-24-0296)
Supplement: Supplementary Figure S8 — Figure S8. (A-C) Pathway analysis for seclidemstat regulated genes in 9 FET-rearranged cell lines visualized with a dot plot using (A) MSigDB curated gene sets (B) gene ontology biological process, and (C) gene ontology molecular function gene signatures. [file crc-24-0296_supplementary_figure_s8_suppsf8.pdf]

Supplementary Figure 8

A

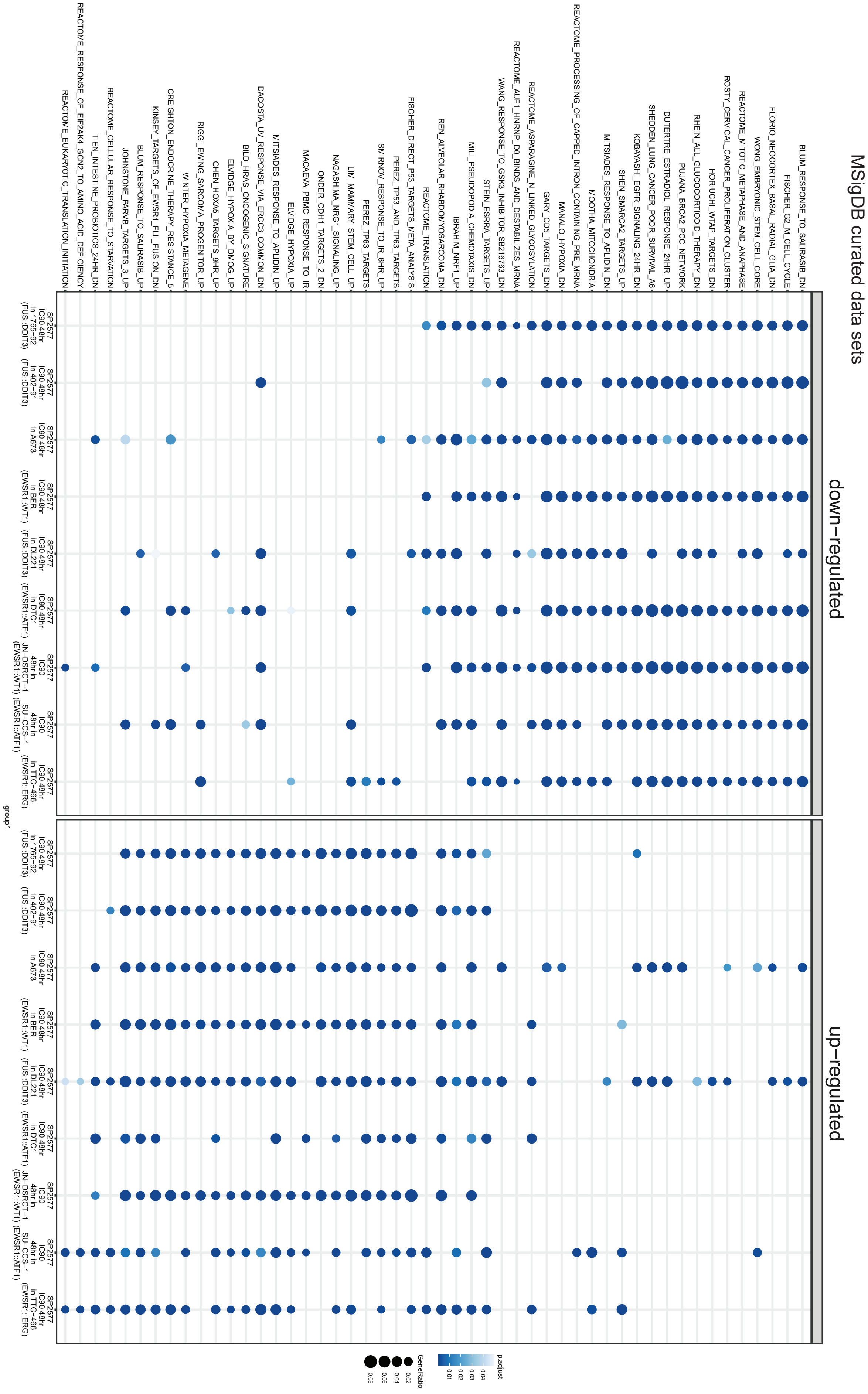

Supplementary Figure 8 (cont.)

B

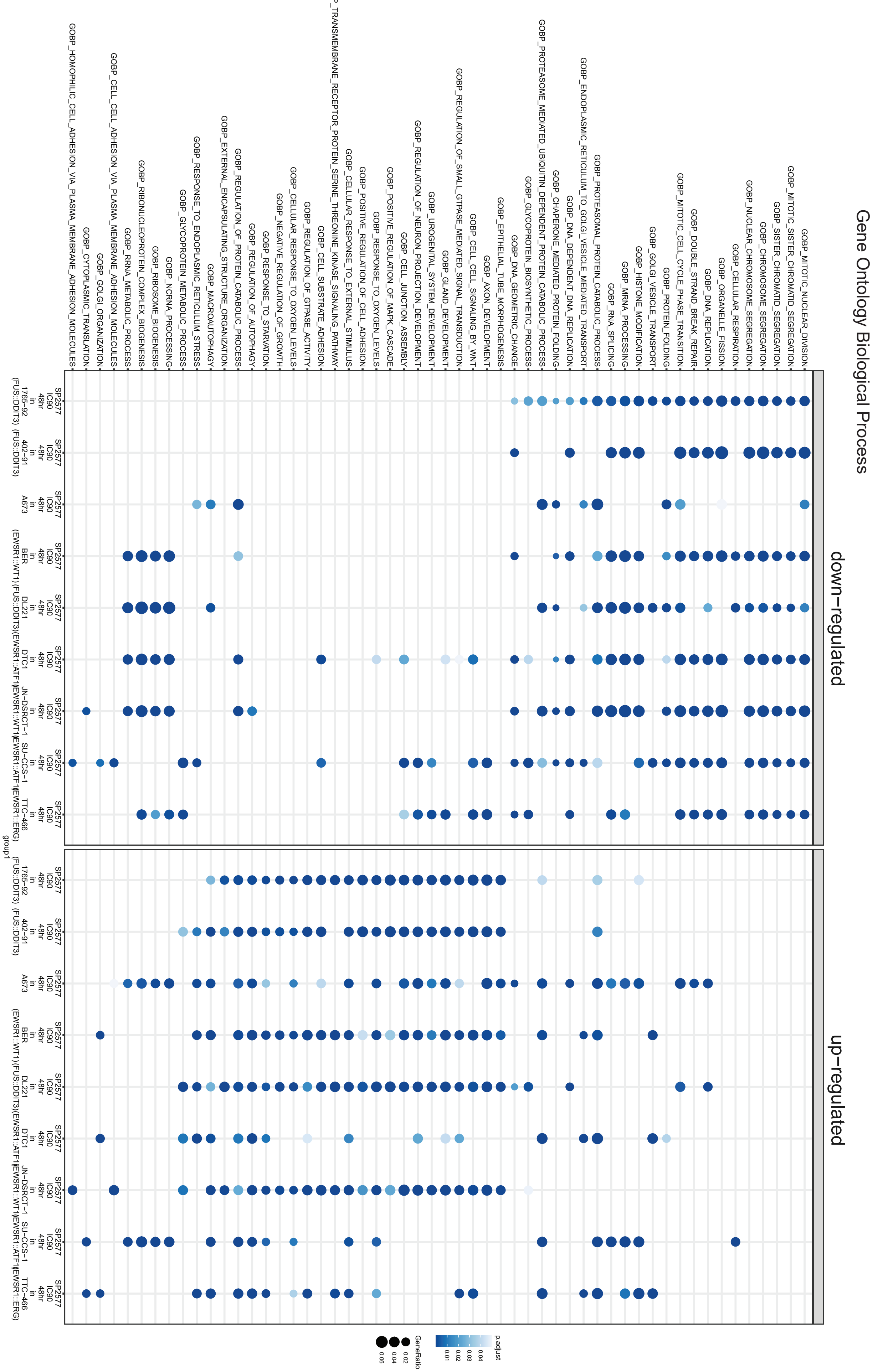

Supplementary Figure 8 (cont.)

C

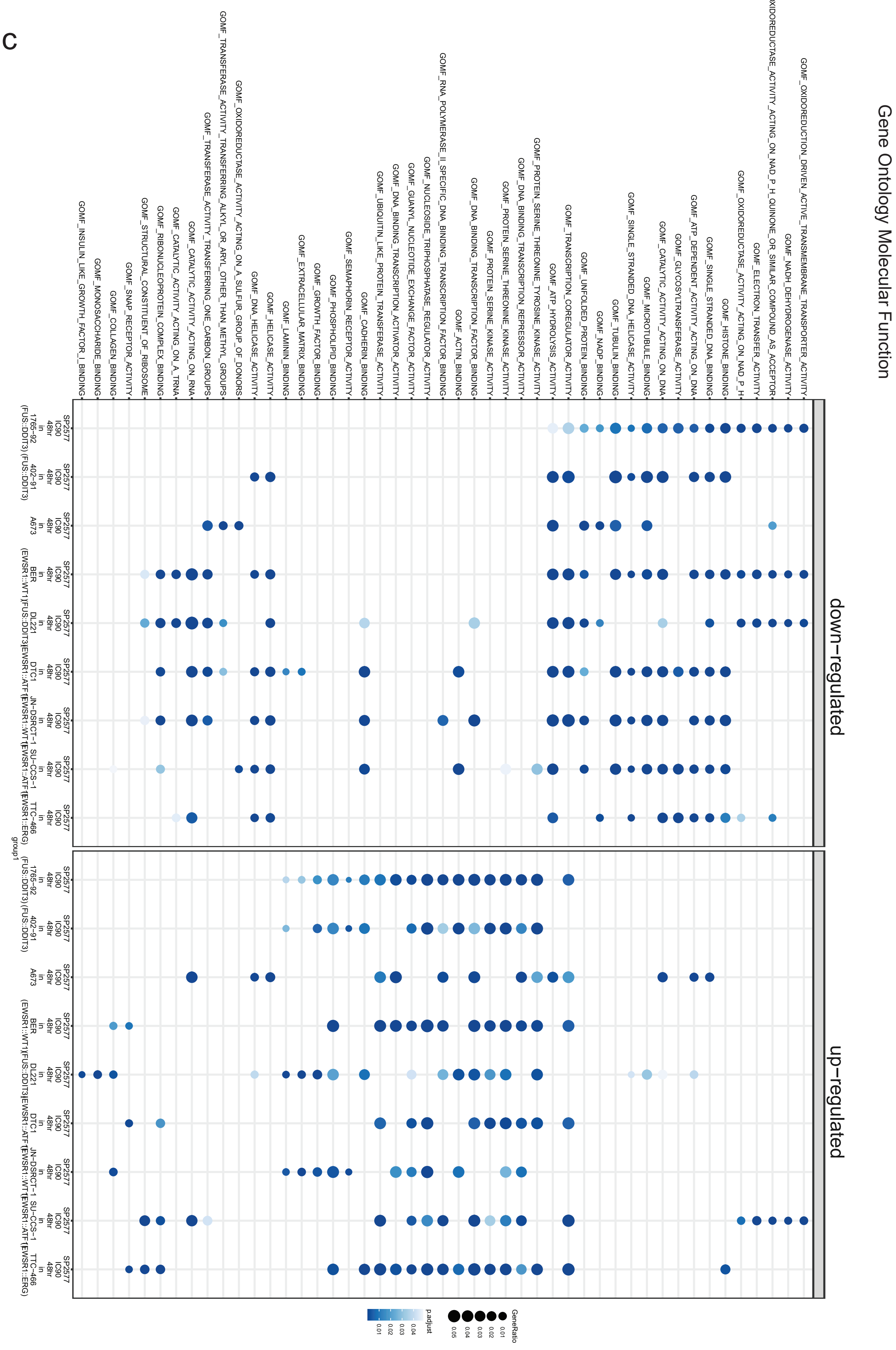

Supplementary Figure 8. (A-C) Pathway analysis for seclidemstat regulated genes in 9 FET-rearranged cell lines visualized with a dot plot using (A) MSigDB curated gene sets (B) gene ontology biological process, and (C) gene ontology molecular function gene signatures.
