# Supplementary material for: Seclidemstat (SP-2577) Induces Transcriptomic Reprogramming and Cytotoxicity in Multiple Fusion–Positive Sarcomas
Source: Cancer Res Commun. 2025 Sep 10;5(9):1584–98. doi: 10.1158/2767-9764.CRC-24-0296 (PMC12421227; doi:10.1158/2767-9764.CRC-24-0296)
Supplement: Supplementary Figure S9 — Figure S9. Venn overlap analysis of SP-2509 (A) up- and (B) downregulated genes in fusion-positive rhabdomyosarcoma cell lines with the Jaccard index and p-values of overlap shown in (C). [file crc-24-0296_supplementary_figure_s9_suppsf9.pdf]

Supplementary Figure 9

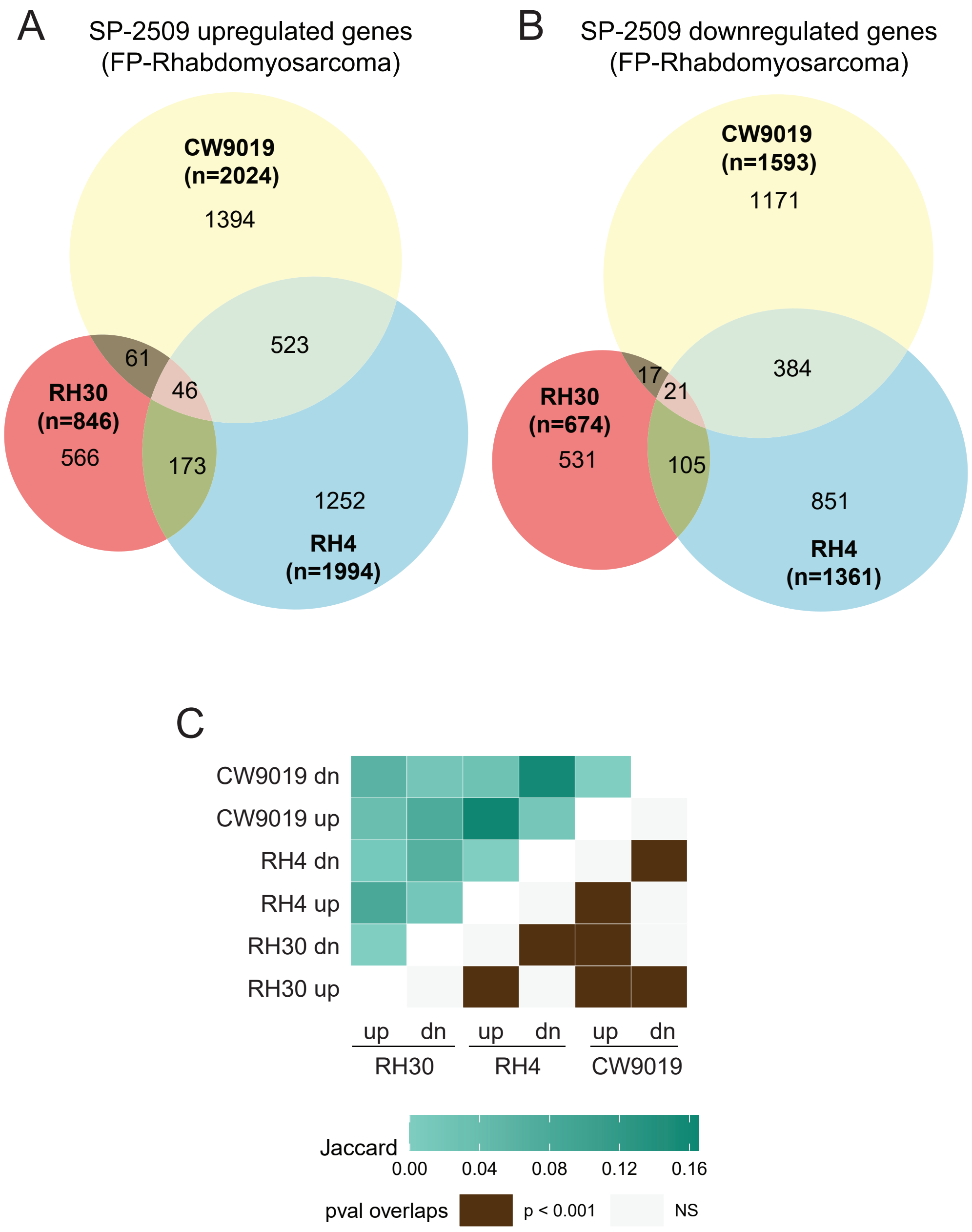

Supplementary Figure 9. (A-C) Venn overlap analysis of SP-2509 (A) up- and (B) downregulated genes in fusion-positive rhabdomyosarcoma cell lines with the Jaccard index and p-values of overlap shown in (C).
