# Supplementary material for: Seclidemstat (SP-2577) Induces Transcriptomic Reprogramming and Cytotoxicity in Multiple Fusion–Positive Sarcomas
Source: Cancer Res Commun. 2025 Sep 10;5(9):1584–98. doi: 10.1158/2767-9764.CRC-24-0296 (PMC12421227; doi:10.1158/2767-9764.CRC-24-0296)
Supplement: Supplementary Figure S10 — Figure S10. Pathway analysis for N’-(2-hydroxybenzilidene)hydrazide regulated genes in all tested cell lines visualized with a dot plot using (A) MSigDB curated gene sets (B) gene ontology biological process, and (C) gene ontology molecular function gene signatures. [file crc-24-0296_supplementary_figure_s10_suppsf10.pdf]

Supplementary Figure 10

A

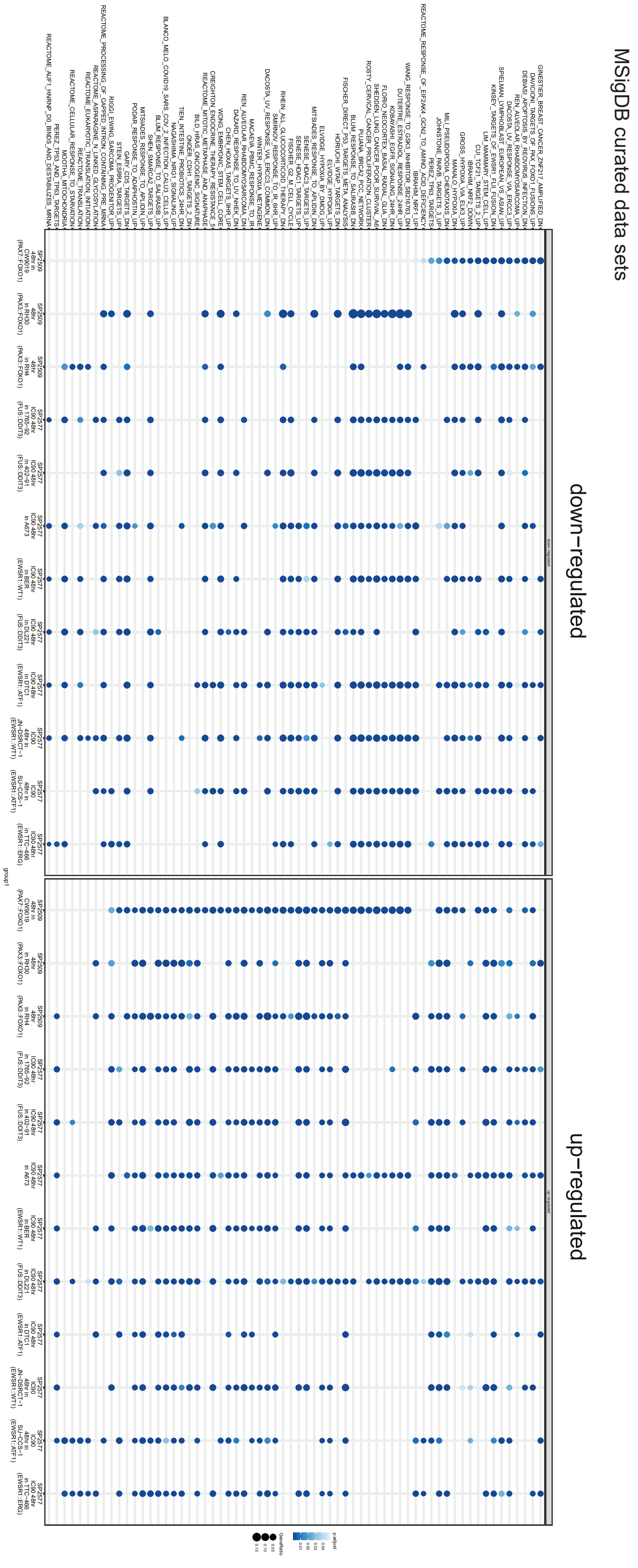

Supplementary Figure 10

B

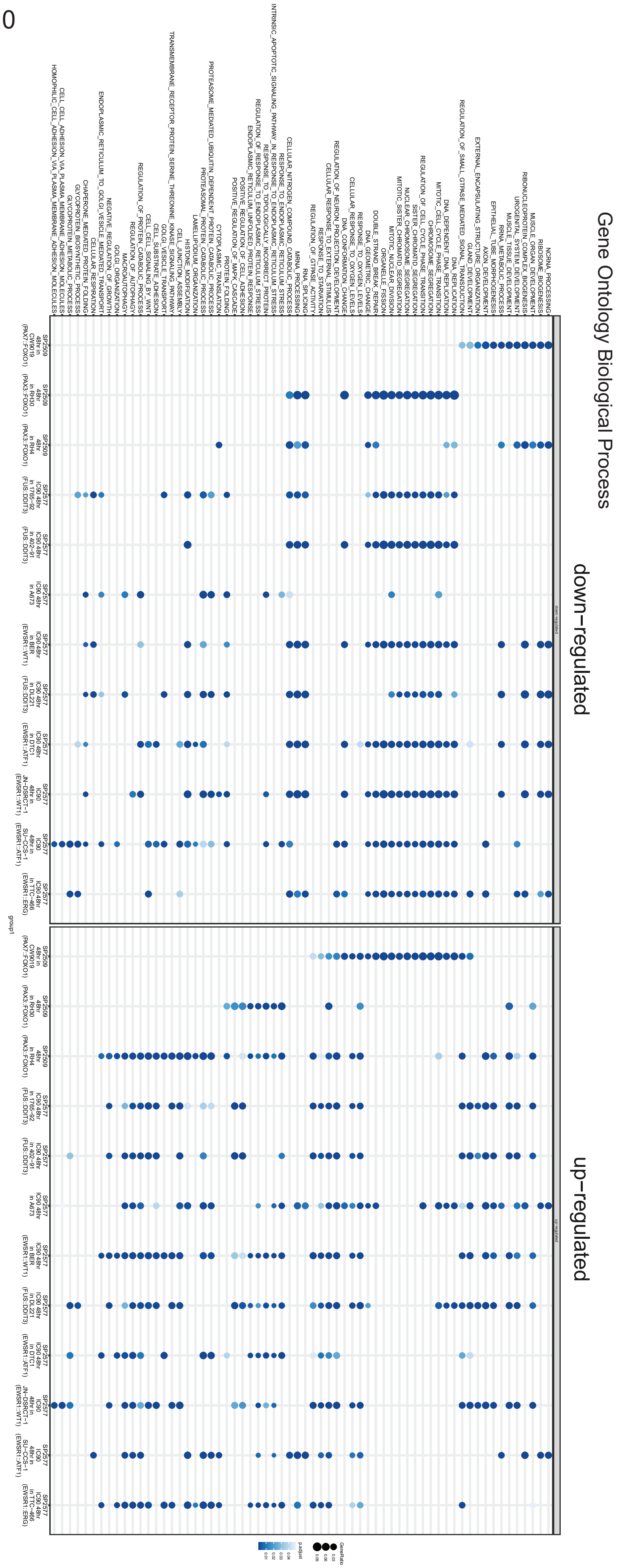

Supplementary Figure 10

C

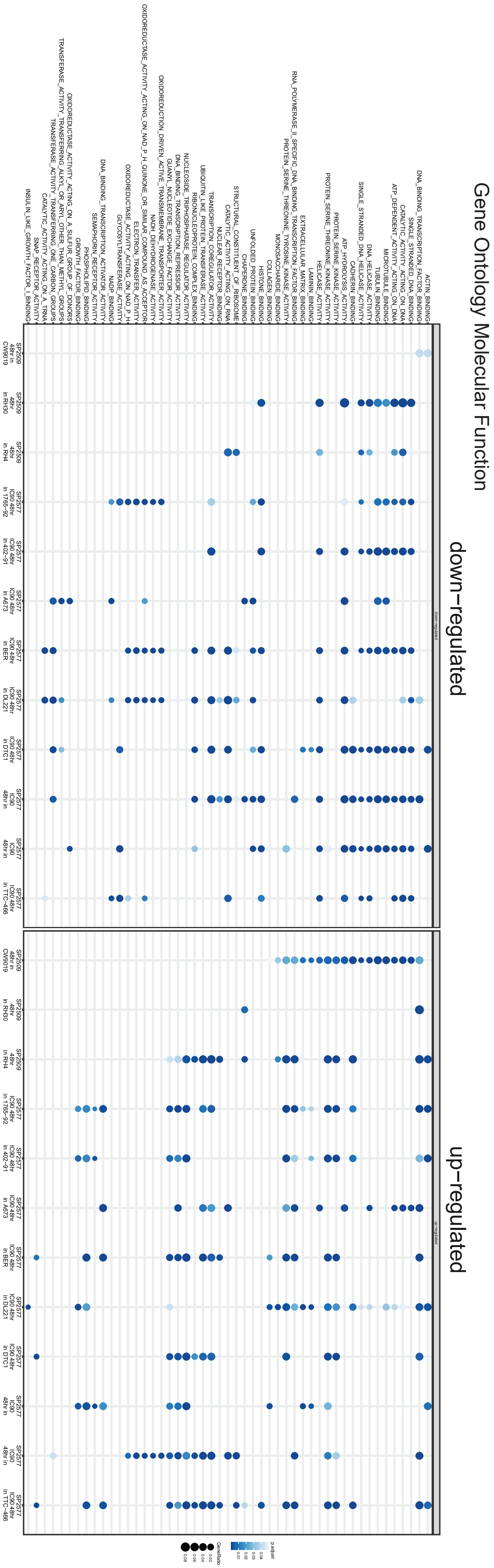

Supplementary Figure 10. (A-C) Pathway analysis for *N'*-(2-hydroxybenzilidene)hydrazide regulated genes in all tested cell lines visualized with a dot plot using (A) MSigDB curated gene sets (B) gene ontology biological process, and (C) gene ontology molecular function gene signatures.
