# Supplementary material for: Seclidemstat (SP-2577) Induces Transcriptomic Reprogramming and Cytotoxicity in Multiple Fusion–Positive Sarcomas
Source: Cancer Res Commun. 2025 Sep 10;5(9):1584–98. doi: 10.1158/2767-9764.CRC-24-0296 (PMC12421227; doi:10.1158/2767-9764.CRC-24-0296)
Supplement: Supplementary Figure S11 — Figure S11. Principal component 2 on the y-axis is plotted against principal component 1 on the x-axis. Different cell conditions are depicted in different colors and different replicates are represented with different shapes. [file crc-24-0296_supplementary_figure_s11_suppsf11.pdf]

Supplementary Figure 11

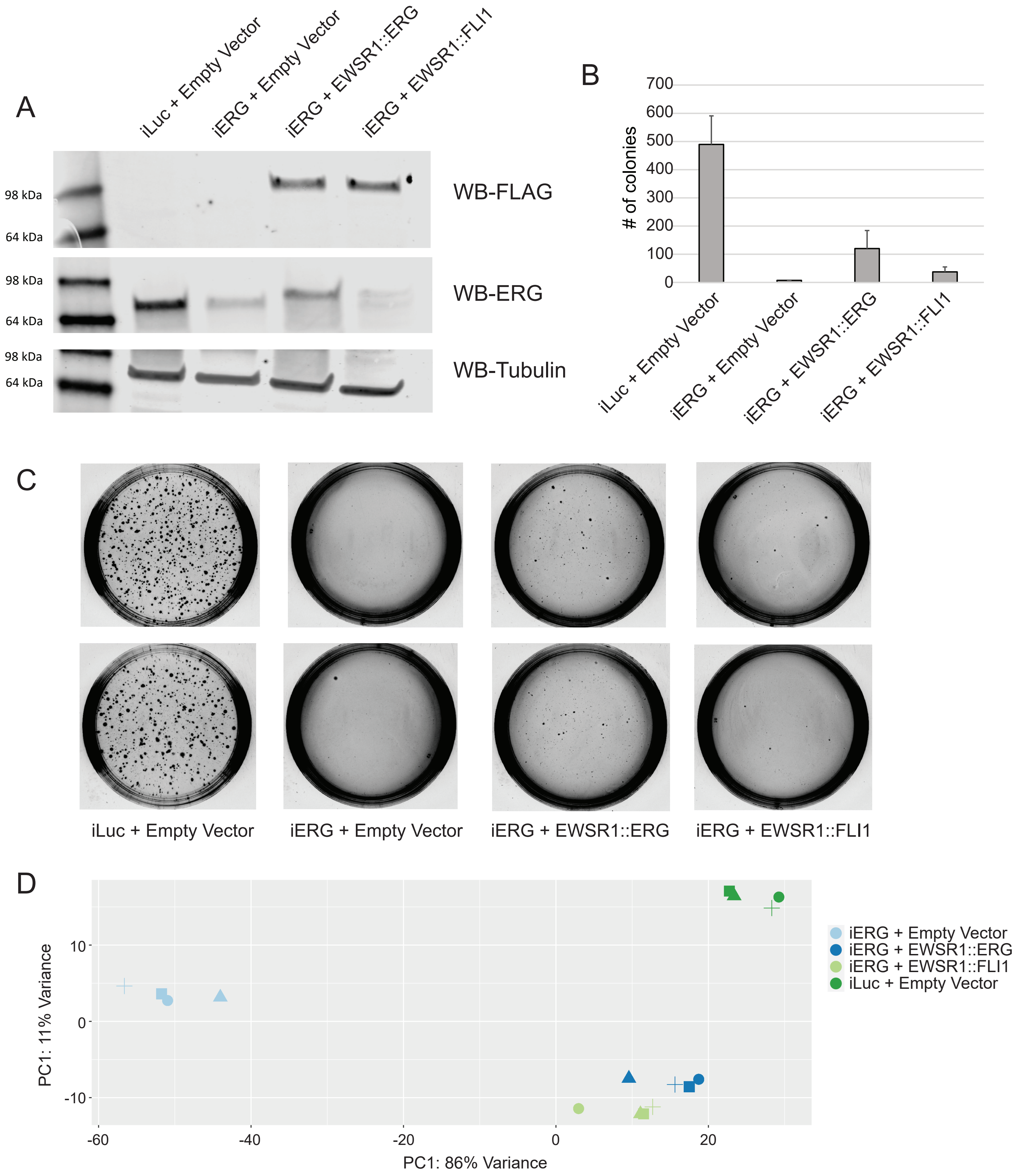

Supplementary Figure 11. (A) Western blot showing depletion of endogenous EWSR1::ERG protein in TTC-466 cells and rescue with either empty vector, 3X-FLAG-tagged EWSR1::ERG, or 3X-FLAG-tagged EWSR1::FLI1 in cells used for downstream agar and RNA-seq assays. Whole cell lysates were used. The 3X-FLAG tag causes a slight upward shift in the observed molecular weight of the rescue constructs. (B,C) (B) Quantification of soft agar assays with representative agar replicates shown in (C). (D) Principal component analysis of gene expression in cells with either endogenous EWSR1::ERG (iLuc+empty vector), EWSR1::ERG1 depletion (iERG+empty vector), or rescue of EWSR1::ERG depletion with either 3X-FLAG EWSR1::ERG (iERG+EWSR1::ERG) or 3X-FLAG EWSR1::FL1 (iERG+EWSR1::FLI1). Principal component 2 on the y-axis is plotted against principal component 1 on the x-axis. Different cell conditions are depicted in different colors and different replicates are represented with different shapes.
