# Supplementary material for: Seclidemstat (SP-2577) Induces Transcriptomic Reprogramming and Cytotoxicity in Multiple Fusion–Positive Sarcomas
Source: Cancer Res Commun. 2025 Sep 10;5(9):1584–98. doi: 10.1158/2767-9764.CRC-24-0296 (PMC12421227; doi:10.1158/2767-9764.CRC-24-0296)
Supplement: Supplementary Figure S12 — Figure S12. Venn overlap analysis of (A) seclidemstat downregulated genes and EWSR1::WT1 activated genes and (B) seclidemstat upregulated genes and EWSR1::WT1 repressed genes in JN-DSRCT-1 cells with the Jaccard index and p-values of overlap shown in (C). (D) Heatmap analysis showing the effect of seclidemstat treatment on the EWSR1::WT1 transcriptional signature. Each row represents a differentially expressed gene (adjusted p < 0.05) and each column is a separate biological replicate. (E-G) Venn overlap analysis of (E) seclidemstat downregulated genes and EWSR1::ERG activated genes and (F) seclidemstat upregulated genes and EWSR1::ERG repressed genes in TTC-466 cells with the Jaccard index and p-values of overlap shown in (G). (H) Heatmap analysis showing the effect of seclidemstat treatment on the EWSR1::ERG1 transcriptional signature. Each row represents a differentially expressed gene (adjusted p < 0.05) and each column is a separate biological replicate. [file crc-24-0296_supplementary_figure_s12_suppsf12.pdf]

Supplementary Figure 12

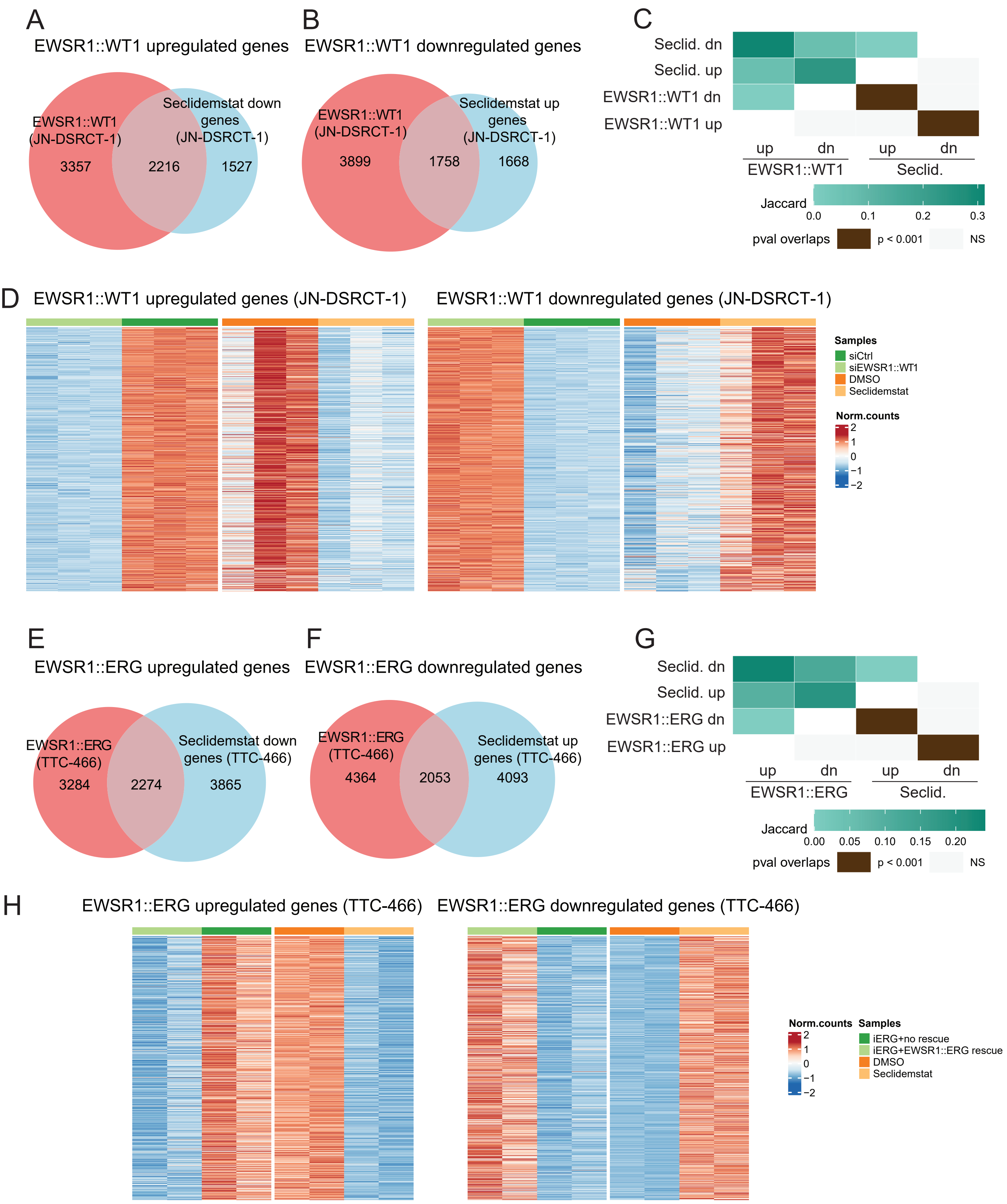

Supplementary Figure 12. (A-C) Venn overlap analysis of (A) seclidemstat downregulated genes and EWSR1::WT1 activated genes and (B) seclidemstat upregulated genes and EWSR1::WT1 repressed genes in JN-DSRCT-1 cells with the Jaccard index and p-values of overlap shown in (C). (D) Heatmap analysis showing the effect of seclidemstat treatment on the EWSR1::WT1 transcriptional signature. Each row represents a differentially expressed gene (adjusted $p<0.05$) and each column is a separate biological replicate. (E-G) Venn overlap analysis of (E) seclidemstat downregulated genes and EWSR1::ERG activated genes and (F) seclidemstat upregulated genes and EWSR1::ERG repressed genes in TTC-466 cells with the Jaccard index and p-values of overlap shown in (G). (H) Heatmap analysis showing the effect of seclidemstat treatment on the EWSR1::ERG1 transcriptional signature. Each row represents a differentially expressed gene (adjusted $p<0.05$) and each column is a separate biological replicate.
