# Supplementary material for: Seclidemstat (SP-2577) Induces Transcriptomic Reprogramming and Cytotoxicity in Multiple Fusion–Positive Sarcomas
Source: Cancer Res Commun. 2025 Sep 10;5(9):1584–98. doi: 10.1158/2767-9764.CRC-24-0296 (PMC12421227; doi:10.1158/2767-9764.CRC-24-0296)
Supplement: Supplementary Figure S13 — Figure S13. Matrices showing GSEA results for various treatments on direct targets of (A) EWSR1::FLI1, (B) EWSR1::ATF1, and (C) EWSR1::WT1. Direct target gene sets were previously published (Refs. 6, 50, and 51). Briefly direct targets were defined as those genes 1) near a fusion protein bound locus or fusion-mediated chromatin loop and 2) with differential expression upon genetic depletion of the fusion. The coloring of each box represents the p-value for that GSEA analysis and the normalized enrichment score is reported inset in each box. [file crc-24-0296_supplementary_figure_s13_suppsf13.pdf]

Supplementary Figure 13

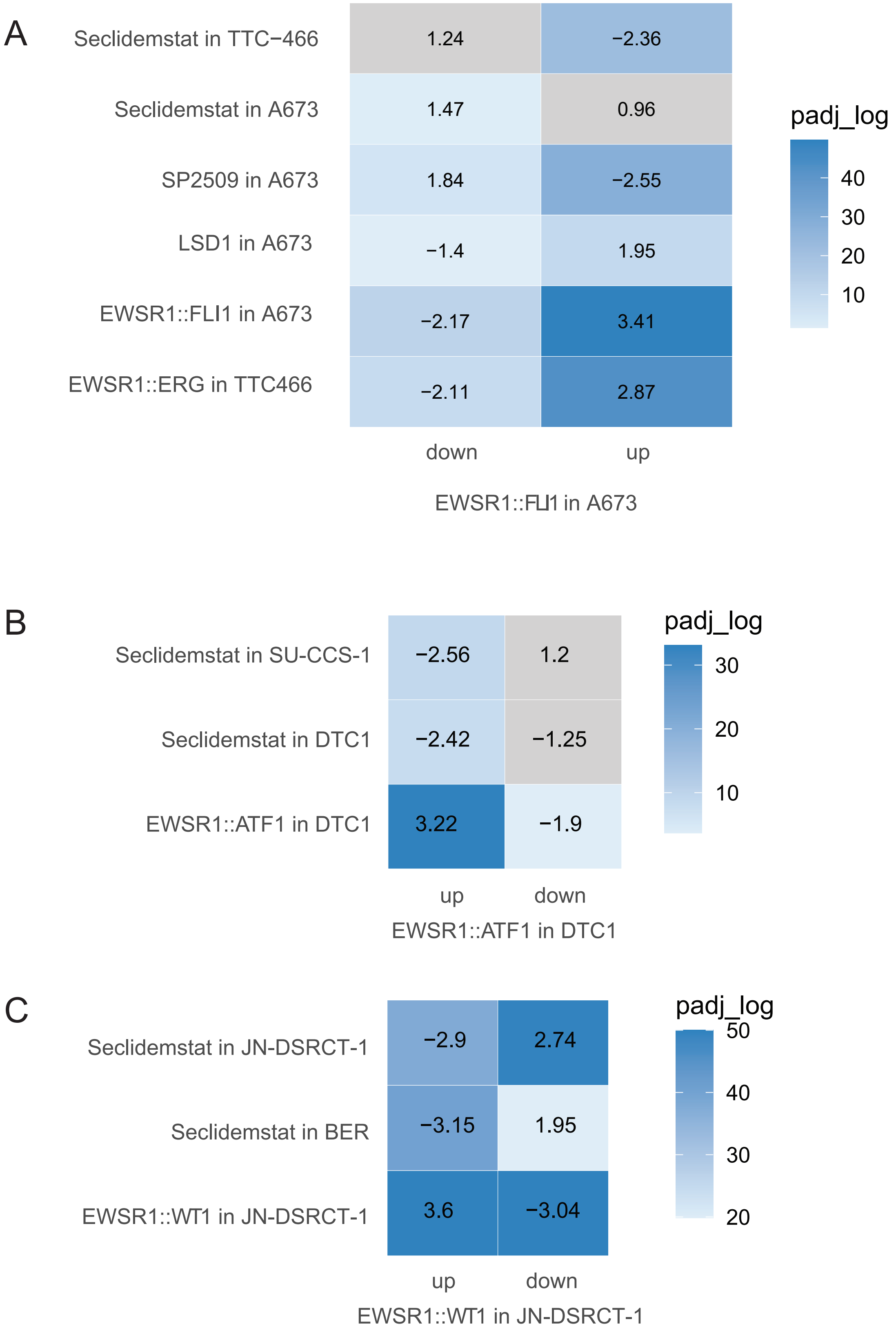

Supplementary Figure 13. (A-C) Matrices showing GSEA results for various treatments on direct targets of (A) EWSR1::FLI1, (B) EWSR1::ATF1, and (C) EWSR1::WT1. Direct target gene sets were previously published (Refs. 6, 50, and 51). Briefly direct targets were defined as those genes 1) near a fusion protein-bound locus or fusion-mediated chromatin loop and 2) with differential expression upon genetic depletion of the fusion. The coloring of each box represents the p-value for that GSEA analysis and the normalized enrichment score is reported inset in each box.
